# Sex differences in musculoskeletal injury and disease risks across the lifespan: Are there unique subsets of females at higher risk than males for these conditions at distinct stages of the life cycle?

**DOI:** 10.3389/fphys.2023.1127689

**Published:** 2023-04-11

**Authors:** David A. Hart

**Affiliations:** Department of Surgery, Faculty of Kinesiology and McCaig Institute for Bone & Joint Health, University of Calgary, Calgary, AB, Canada

**Keywords:** sex differences, sex hormones, heterogeneity within a sex, molecular mechanisms, hormone receptors, epigenetics

## Abstract

Sex differences have been reported for diseases of the musculoskeletal system (MSK) as well as the risk for injuries to tissues of the MSK system. For females, some of these occur prior to the onset of puberty, following the onset of puberty, and following the onset of menopause. Therefore, they can occur across the lifespan. While some conditions are related to immune dysfunction, others are associated with specific tissues of the MSK more directly. Based on this life spectrum of sex differences in both risk for injury and onset of diseases, a role for sex hormones in the initiation and progression of this risk is somewhat variable. Sex hormone receptor expression and functioning can also vary with life events such as the menstrual cycle in females, with different tissues being affected. Furthermore, some sex hormone receptors can affect gene expression independent of sex hormones and some transitional events such as puberty are accompanied by epigenetic alterations that can further lead to sex differences in MSK gene regulation. Some of the sex differences in injury risk and the post-menopausal disease risk may be “imprinted” in the genomes of females and males during development and sex hormones and their consequences only modulators of such risks later in life as the sex hormone milieu changes. The purpose of this review is to discuss some of the relevant conditions associated with sex differences in risks for loss of MSK tissue integrity across the lifespan, and further discuss several of the implications of their variable relationship with sex hormones, their receptors and life events.

## 1 Introduction

A large number of diseases or conditions of the musculoskeletal (MSK) system exhibit sex differences. Some of these diseases and conditions occur at different stages across the lifespan, prior to the onset of puberty, after onset of puberty, and in the female population, in the post-menopausal state. While many of these conditions are idiopathic (the causes are unknown), some are related secondarily to autoimmune diseases and other sex differences are related to differences in injury risk to MSK tissues.

Sex differences have been noted for risk to injure elements of the MSK system, for example, injuries to the anterior cruciate ligament (ACL) of the knee, an injury which is more prevalent in young females engaged in some sports with the female to male ratio of 5/1 in some of them (Harmon and Ireland, 2000; Wild et al., 2012; Edison et al., 2022). Some of the risk for the latter can be traced to changes in joint laxity across the menstrual cycle (Park et al., 2009), while others may be related to muscle function issues between females and males (Hale et al., 2014; Marotta et al., 2019). However, the basis for all injury-related risks that exhibit sex differences in incidence have not been characterized.

For a number of other conditions or diseases, MSK involvement may be secondary to immune dysfunction and subsequent localization within a joint (generally or involving specific joints). As many autoimmune diseases affect more females than males, this means that such conditions that affect joints and other connective tissues are mainly females. An example of such a scenario is rheumatoid arthritis (RA) which can be initiated pre-puberty (i.e., Juvenile Idiopathic Arthritis; Juvenile Inflammatory Arthritis; JIA) (Glass and Giannini, 1999; Cattalini et al., 2019), as an adult, or even in post-menopausal females (Serhal et al., 2020; Shah et al., 2020; Sugihara, 2022). JIA exhibits sex differences in incidence with a predilection for females compared to males of 3-6.6/1 (Cattalini et al., 2019). However, sex hormones alone cannot account for the sex disparities given the early age of onset for many patients with JIA. Furthermore, some subsets of patients with JIA exhibit involvement of other tissues that also appear to be associated with sex. Thus, patients with knee involvement and also chronic anterior uveitis appear commonly in females with JIA (Angeles-Han et al., 2013; Cattalini et al., 2019).

A third category of MSK conditions that exhibit sex differences in incidence/prevalence are those not associated with any overt linkage to immune dysfunction or obvious injury event. An example of such a condition is adolescent idiopathic scoliosis, a condition affecting 1%–4% of adolescents (Penha et al., 2018; Yan et al., 2020), and a condition resulting in spinal deformities of varying extent, with onset in the post-puberty environment of growth and maturation (Chung et al., 2020; Liang et al., 2021). It occurs mostly in young females (Chung et al., 2020; Yan et al., 2020; Liang et al., 2021), with a F/M ratio of up to 10/1 for those with curvatures of more than 30° (Raggio, 2006). While some cases can be treated with braces, others require surgery to correct alterations that severely impair both function and quality of life for these young individuals (Diarbakerti et al., 2018; Burger et al., 2019; Cheung et al., 2019). While progression of scoliosis may slow after skeletal maturity, in a subset of individuals it may “re-activate” following menopause and require surgery latter in life (discussed below).

Other examples of this third category are development of osteoarthritis (OA), osteoporosis (OP), and sarcopenia in post-menopausal females [reviewed in 21]. In ages prior to menopause, the incidence of “idiopathic” (no cause known) osteoarthritis in females and males is ∼1:1 but after menopause the ratio becomes >2:1 [discussed in 21]. While osteoporosis can occur in younger individuals due to drug treatment and some surgeries, the vast majority of osteoporosis patients develop their disease after menopause. In the post-menopausal age bracket, approximately 70%–75% of OP patients are female and ∼25% are male. However, even within the OP patient population, the rate of bone loss is quite variable so there is heterogeneity within the disease as well. Sarcopenia, or loss of muscle and muscle function can also occur with aging and contribute to loss of “muscle-bone” unit function (Hart et al., 2021; Hart, 2022a).

From the discussion above there are a number of points that emerge regarding sex differences and MSK disease risk, and these are detailed in point form.

1) Sex differences are relative and not absolute, with only a subset of the total population affected.
2) Sex differences in MSK conditions can be evident during hormone level changes at their onset (puberty) and loss (menopause), as well as during development. Thus, MSK conditions are evident across the lifespan, and often occur during or as a result of the transition phases such as puberty and menopause.
3) Heterogeneity within a sex is evident for risk, so factors other than just sex hormones are likely contributing to risk.
4) Different MSK conditions exhibiting sex differences likely involve different molecular mechanisms, and not all can be directly traced to sex hormones.
5) MSK involvement in diseases exhibiting sex differences may be primary or secondary

The purpose of this review is to discuss in some detail a limited but representative spectrum of MSK system diseases and conditions with regard to onset and progression that exhibit sex-dependent differences. The goal is to attempt to better understand similarities and differences in the molecular mechanisms involved, and their potential relationship to sex hormones and sex hormone receptors, across the lifespan. As the number of MSK diseases and conditions that exhibit sex differences is quite large, the subset chosen for discussion represent different tissues, different phases of the life cycle, and may represent a number of possible mechanisms being involved. As will be discussed, studies of sex differences and the role of sex hormones and their receptors is complex, due in part to human heterogeneity and the circumstances around specific stages of the life cycle.

## 2 Sex differences in risk for injury to MSK tissues during development, growth and maturation

### 2.1 Neonatal hip dysplasia: Development

Sex differences in risk for MSK dysfunction can arise during fetal development. An example of this category is hip dysplasia in babies when they are born. Many studies have indicated that the incidence of neonatal hip dysplasia is greater in females than males, with an Odds Ratio for F/M of 3.8-4 commonly reported (de Hundt et al., 2012; de Oliveira Barbosa and Albernaz, 2019). Within this population there are some features that have been raised and risk factors identified including family history and genetics (Shaw et al., 2016; Swarup et al., 2018; Harsanyi et al., 2020; Zhang et al., 2020). The incidence of unilateral hip dysplasia is greater on the left side than the right, and only a small subset (∼10%) of patients exhibit bilateral hip dysplasia (Mousavibaygei et al., 2022).

When detected this condition can often be corrected by non-invasive procedures using braces or a Pavlik harness (Vafaeian et al., 2019) to maintain the ball of the hip within the socket. Failure to correct the condition early after birth leads to an inability to treat conservatively and development of early OA, often requiring arthroplasty at an early age (Shaw et al., 2016). Thus, to maintain proper biomechanics for the hip during subsequent growth and maturation requires proper placement of the two parts of the joint. Interestingly, whether excessive hip laxity develops in this population after puberty, when sex hormone levels rise and cycle during the menstrual cycle, could not be found in the literature. If tissues of individuals experiencing developmental hip dysplasia had some intrinsic unique responsiveness to sex hormones, one might expect a possible resurgence of the hip laxity leading to altered biomechanics and risk for early OA.

Mechanistically, the basis for sex-dependent differences in development of hip dysplasia is not well defined. As the non-invasive treatment of the condition is usually a temporary and short-term intervention to keep the hip in the hip socket, the hip dysplasia is not a permanent developmental problem and once removed from the intrauterine environment, the neonate can “correct” the deficiency. As ligaments support the stability of the head of the femur in the hip socket, it was once thought that the ligamentum teres was relevant to the developmental problem. However, the gross characteristics of this ligament were not found to be significantly different in patients and controls (Walker, 1980). Such studies did not characterize the functioning of such ligaments so perhaps the laxity of connective tissues contributing to the integrity of hip development were unduly affected by maternal hormones, a situation that self corrected after birth under conditions counteracting the pre-birth structural influence. Of note and potentially relevant to this point, elevated levels of estrogen receptors have been detected in hip tissues from babies with developmental dysplasia of the hip (Desteli et al., 2013), as well as higher levels of receptors for relaxin (an IGF-1 related protein) elevated late in pregnancy and in milk in some species (Steinmetz et al., 1987; Goldsmith et al., 1994) including humans (Schauberger et al., 1996), and in hip tissues from babies with this dysplasia (Ayanoglu et al., 2021). However, this interpretation would not however explain any left-right hip differences! As the hip dysplasia can vary in extent (i.e., severity), there must also be factors other than hormone levels involved, possibly at the level of cells within the tissues, at the level of sex-hormone receptors, or possibly genetic factors (Harsanyi et al., 2020). Thus, any left-right differences within a sex may have other variables involved in the outcomes. This conclusion would also be supported by results from pregnant humans where joint laxity occurs but does not correlate with serum relaxin levels (Schauberger et al., 1996).

Of note, there has been continual improvement in the detection of hip dysplasia using imaging modalities (Barrera et al., 2019; Ghasseminia et al., 2021; Kilsdonk et al., 2021) so detection of cases has experienced increased sensitivity (Zonobi et al., 2018). Such approaches may also shed light on sex differences in patients with borderline dysplasia (Saks et al., 2021). Further improvements may be possible with enhanced technology using machine learning and artificial intelligence approaches and improved training (Ghasseminia et al., 2022). Further, some reports also indicate it may be possible to detect the condition in the prenatal period (Komut and Zehir, 2021). These will be important advances to continue as even the more subtle variants of hip dysplasia could have impact for development of hip osteoarthritis later in life and potentially explain the female preponderance of osteoarthritis in the post-menopausal phase of the lifespan (Hart et al., 2021). This is relevant as even if detected in childhood and treated, many patients still exhibit altered gait patterns (Lee et al., 2022a).

Interesting, the concept of ligament laxity playing a role in developmental hip dysplasia perhaps should not be dismissed as ligament laxity may also play a role in risk for knee injuries during adolescence and beyond as discussed below. It is also of interest to determine whether females with neonatal hip dysfunction would also predispose for development of hip issues following pregnancies where laxity again occurs and could again impact the integrity of the hip biomechanics (discussed below).

Some individuals, mainly females again, are diagnosed with acetabular dysplasia as adolescents or adults (Lee et al., 2013a). Such dysplasia also shows a left hip susceptibility and can lead to osteoarthritis. While very young babies with hip instability can have acetabular dysplasia (Wenger et al., 2013), some authors suggest that acetabular dysplasia is a separate disease from development dysplasia (Lee et al., 2013a). Such suggestions will require further investigation as to the underlying mechanisms, but both conditions appear to exhibit some sex differences.

### 2.2 Sex differences in risk for Juvenile Idiopathic Arthritis

Juvenile idiopathic arthritis (JIA) can develop in very young children through to skeletal maturity. Thus, this autoimmune disease affecting the joints of growing individuals can occur prior to onset of puberty as well as after puberty (Barnes et al., 2010; Hollenbach et al., 2010; Ahmed et al., 2014; Cattalini et al., 2019). JIA exhibits a sex bias of F/M = 3-6.6/1 (Cattalini et al., 2019) although this ratio may be dependent on race and ethnicity variables (Ringold et al., 2013; Ahmed et al., 2014). In addition, the sex bias cannot be explained by sex hormones alone (Chiaroni-Clarke et al., 2016). Some JIA patients also develop psoriatic arthritis (∼5%) (Brunello et al., 2022), while others develop uveitis (inflammation of the anterior chamber of the eye) which can lead to blindness if not treated (Rosenberg, 1987; Rosenberg, 2002; Quartier, 2021). A subset of females with JIA are at particular risk to develop uveitis (Haasnoot et al., 2019; Lee et al., 2019). Interestingly HLA-B27 is reported to be a predictor of some disease characteristics in boys (Berntson et al., 2008). Therefore, JIA is likely an umbrella term for a heterogeneous disease with different variables influencing the sex differences in the disease and its sequalae.

While much of the research on JIA has focused on immune aspects of the disease or characterization of clinical presentation differences (Hinks et al., 2018; Eng et al., 2019; Gohar et al., 2019; Nigrovic et al., 2021; Onel et al., 2021), details regarding why certain joints are involved in the disease process and not others, why the eye is involved in some patients, and how the sex differences are manifested in pre-puberty *versus* post-puberty cases remains to be elucidated. As reported by Kuntze et al. (2020), the presence of JIA affecting the knees can impact gait during growth and development which may have long-lasting implications. While in a preclinical model, it has been reported by Kydd et al. (2007) that induction of an inflammatory arthritis in the female rabbit knee can lead to molecular changes in the eye. Additional unpublished results indicate that induction of unilateral knee arthritis in this model leads to changes in the cornea and vitreous humor of both eyes [Kydd, Hewitt and Hart, unpublished observations]. This led to the concept that there may be a “knee-eye-brain axis” of regulation that is mediated either by humoral factors or neural mechanisms, and this may be relevant to the JIA-uveitis link (Hart, 2018). However, such an axis would not explain the relative rarity of uveitis in adult rheumatoid arthritis patients, but the relatively common occurrence of non-infectious uveitis in adults with spondyloarthritis, as mainly unilateral uveitis (Seve et al., 2015).

### 2.3 Adaptations during growth and maturation: Puberty

#### 2.3.1 Sex differences in lower extremity maturation characteristics during the puberty transition: Gait

During the early growth phase, the lower extremity motion segments appear to grow in a patterned manner, but considerable individual variation in rate can occur as well as parameters such as varus-valgus features, alignment, and integration of the various components that may grow at different rates (Weir, 1991; Hart et al., 2002). At the onset of puberty additional growth rate variation can occur as well as the appearance of sex-dependent features. Males appear to develop more muscles and their growth rates can vary, but a rapid growth rate can lead to development of dysregulated growth and such dysregulation can lead to conditions such as Osgood-Schlatter disease which mainly affects males (Monasterio et al., 2021; Neuhaus et al., 2021; Gaulrapp and Nuhrenborger, 2022). In a recent clinical series with 126 participants, 101 were males (Gaulrapp and Nuhrenborger, 2022). While some cases of Osgood-Schlatter disease in the knee can occur as a result of overuse *via* participation in sport, it is not always the case (Gaulrapp and Nuhrenborger, 2022). The condition is usually self-limiting and resolves naturally (Monasterio et al., 2021; Neuhaus et al., 2021; Gaulrapp and Nuhrenborger, 2022).

As assessed by walking, aspects of gait appear to be influenced by puberty, likely influenced by puberty-associated alterations in use of particular muscles (Di Nardo et al., 2017) in the context of the integrated functioning of the lower extremity motion segments comprised of the bones, muscles and joints (hip, knee and ankle. Interestingly, these effects are most prominent in females, and the changes are finally set at the time of skeletal maturity when growth and maturation are complete (Di Nardo et al., 2017).

In addition, both males and females land after a jump similarly prior to the onset of puberty, but land quite differently post-puberty (Quatman et al., 2006; Hewett et al., 2015a), and this can pose risks for knee injury (Hewett et al., 2015b). At skeletal maturity, the set points for the joints of a lower extremity motion segment are fairly well established and such set points may provide functioning during adult life. However, some of the variation in how this set point derived from the functional integration of contributing tissues is established may pose risk during the aging process when the integrative nature becomes less functional, such as loss of muscle integrity resulting from less physical activity by 60 years of age or following the onset of sarcopenia (Kaji, 2013). Some of the variation, such as malalignment, could exert more potential risk than some other configurations (Nam et al., 2020; Springer et al., 2020; Black and Clark, 2022). Thus, some sex-dependent developmental changes occurring after onset of puberty could contribute to joint disease development later in life, particularly after menopause in females.

#### 2.3.2 Joint laxity and the menstrual cycle

With the onset of puberty, females begin to cycle and after a period of time, such menstrual cycles become fairly regular in length. Accompanying those cycles are changes in joint laxity such as for the knee (Somerson et al., 2019; Maruyama et al., 2021; Maruyama et al., 2022). However, such findings have not been consistent across studies, potentially due to the finding that ∼20% of young skeletally mature females do not experience changes to joint laxity at different stages of the menstrual cycle (Park et al., 2009). Why this 20% of females do not exhibit joint laxity is not clear as their hormone levels across the menstrual cycle do not differ from those individuals who do experience changes in joint laxity (Park et al., 2009). As changes in joint laxity are reported to be associated with risk for knee injury, specifically ACL ruptures (Somerson et al., 2019), it is still unknown whether this non-responsive subset of females is at less risk for such injuries. However, one can conclude that heterogeneity in the regulation of joint biomechanics is apparent after onset of puberty and established at skeletal maturity. Whether this response/non-response phenotype variation is a biomarker for development of knee OA later in life is currently unknown.

#### 2.3.3 Sex differences in risk for ACL rupture post-puberty

Sex differences have been observed for a number of sports injuries either before or after skeletal maturity (Lin et al., 2018). However, injuries to the anterior cruciate ligament (ACL) of the knee are very common, particularly among young athletes and athletes in general with over 200,000 per year in the United States (Musahl and Karlsson, 2019). Most of these are non-contact injuries, particularly in sports that require cutting maneuvers (Renstrom et al., 2008; Musahl and Karlsson, 2019). Interestingly, females are more likely to suffer ACL injuries than males on a participation rate index (Ellison et al., 2021), often up to 5/1 in some sports such as soccer (Harmon and Ireland, 2000; Ireland, 2002; Wild et al., 2012; Sutton and Bullock, 2013; Edison et al., 2022). Often, an ACL rupture requires reconstruction to help stabilize the knee, but even with reconstruction the long-term consequence of the injury is to develop osteoarthritis (OA) at an early age, with up to ∼50% of individuals developing OA within 10–15 years post-injury (Lohmander et al., 2007). While some patients appear to be able to cope well to the unreconstructed injury, others do not (Kaplan, 2011). Even after ACL reconstruction surgery, males and females exhibit different gait and muscle patterns (Arhos et al., 2022), and thus, the sex differences persist after the injury. Even after ACL reconstruction, loss of quadricep muscle integrity persists (Gumucio et al., 2018; Johnson et al., 2018) and may contribute to OA risk.

Given the serious impact such ACL injuries have on the patients, and the sex differences, considerable effort has gone into understanding the risk factors for such injuries (Barber-Westin et al., 2009; Lawra et al., 2021; Maniar et al., 2022)and potential prevention strategies (Myklebust et al., 2007; Brophy et al., 2010; Sadoghi et al., 2012; Myklebust et al., 2013; Emery et al., 2015; Zebis et al., 2016; Butler et al., 2022; Mattu et al., 2022; Weingart et al., 2022) to diminish the number of such injuries particularly in females. Some neuromuscular training programs to address muscle imbalances were proven to have significant impact in reducing ACL injuries in Norwegian female handball players, but uptake outside of clinical trials is often low and even when successful in a controlled setting, may not be embraced by the general population (Myklebust et al., 2013). Therefore, even in the presence of scientific and clinical evidence, changing behavior remains a challenge.

#### 2.3.4 Sex differences in risk for development of adolescent onset scoliosis post-puberty

Adolescent idiopathic scoliosis (AIS) is a disease of spinal curvature that usually starts after the onset of puberty. It is defined by a lateral curvature of the spine of at least 10° (Weingart et al., 2022). With increasing severity of the curvature, the ratio of affected females to males increases leading to a ratio of females to males of ∼10/1 for those with curvatures of >30° (Kuznia et al., 2020). Thus, with increasing severity there are more females affected than males. The incidence of AIS is approximately 1%–4% of the adolescent population and the incidence is similar in many countries that are geographically diverse (USA, China, Brazil) (Penha et al., 2018; Yan et al., 2020; Weingart et al., 2022).

A range of treatments for AIS are available and these range from exercises to bracing and finally surgery for those with severe disease and a large degree of deviation (i.e., Cobb angle) (Day et al., 2019; Kaelin, 2020; Dufvenberg et al., 2021; Bowden et al., 2022; Park et al., 2022; Seleviciene et al., 2022; Turner et al., 2022; Zaina et al., 2022). For those patients with severe disease and needing surgery, the implantation of rods and screws can alleviate the curvature-associated complications but can also affect quality of life during post-skeletal maturity. The majority of the curvature occurs prior to skeletal maturity and progression of the curvature appears to slow down after skeletal maturity. Thus, even in the continued presence of cycling hormone levels in females, the progression of the curvature in AIS appears to stabilize at skeletal maturity. No evidence indicating that AIS in males differed from females with regard to disease progression and skeletal maturity was found.

Since the onset of AIS correlated with the inset of puberty and associated hormone level changes and growth rates, some investigators have assessed the effect of subsequent pregnancy on females experiencing AIS as younger individuals. Both Betz et al. (1987) and Dewan et al. (2018) reported minimal effects of AIS on pregnancy and pregnancy outcomes. Therefore, hormonal changes occurring during pregnancy did not overtly impact a “re-activation” of the AIS in the female population.

For coordinated growth following onset of puberty requires that all components of a system work in an integrated manner. Based on such a concept, the concept that muscle imbalances during growth could play a role in development and progression of AIS has been advanced (Pollak et al., 2013; Park et al., 2021; Stepien et al., 2022). Whether such asymmetry of muscles was present before puberty and only exacerbated by puberty and rapid growth has also been raised by Stepien et al. (2022) and also elaborated on regarding potential embryonic origins has been suggested (Burwell et al., 2016). The literature has also been reviewed that indicates that there is a correlation between pectus deformity and AIS (Van Es et al., 2022). Interestingly, analysis of paravertebral muscles has indicated that potentially relevant molecular differences can also be detected with biopsies from AIS patients (Rusin et al., 2012; Kudo et al., 2015). Chmielewska et al. (2020) reported that methylation of an estrogen receptor (ESR 2) occurred more commonly in AIS patients but did not correlate with disease severity. Kudo et al. (2015) reported that expression of nerve growth factor (NGF) and estrogen receptor-alpha was higher in AIS patients, but only levels of NGF correlated with curvature severity. However, other studies indicated estrogen receptor 2 levels in such muscles showed a correlation with Cobb angle in AIS patients (Rusin et al., 2012). Additional studies have explored potential roles for growth related hormones (Skogland and Miller, 1980) and sex hormone levels (Raczkowski, 2007; Kulis et al., 2015) on AIS development and progression. However, this type of data base is not large and likely needs further study.

As discussed previously, the incidence of AIS is only ∼1–4% of the population so there must be other factors or variables than puberty-associated sex and growth-related hormone levels involved. In addition, it is not known whether in detail whether the muscle involvement is primary or secondary regarding causality. To address some of the gaps, a number of studies and reviews have explored and analyzed the possibility that there are genetic or epigenetic-related variables at play in disease initiation and progression (Gorman et al., 2012; Zaydman et al., 2021; De Salvatore et al., 2022; Faldini et al., 2022). A number of studies have implicated a variety of genes and gene families in AIS including Fibrillin-1 (Sheng et al., 2019; De Azevedo et al., 2022), Fibrillin-1 and Fibrillin-2 variants associated with severe disease (Buchan et al., 2014), estrogen receptor variants and polymorphisms (Esposito et al., 2009; Wang et al., 2020), the NUCKS1 gene in Chinese adolescents (Xu et al., 2017), and the helicase DNA-binding protein 7 (CHD7) (Wu et al., 2021; Wu et al., 2022). While additional studies are needed in this area, the heterogeneity in genetic contributions detected thus far may indicate that the term AIS is an umbrella term for multiple subsets of the disease, or more information is needed to better understand the molecular basis for a common set of pathways.

## 3 Influence of sex on MSK tissues as an adult

There is a wide spectrum of conditions that can arise following skeletal maturity that exhibit sex differences in either incidence, characteristics of progression or are restricted to females due to physiologic differences. Thus, the following discussion contains only examples of such conditions and is selective and not meant to be all inclusive.

### 3.1 The menstrual cycle, joint laxity and ACL ruptures

Following attainment of skeletal maturity, many females still experience changes in knee joint laxity during the menstrual cycle and this again was proposed to be associated with increased risk for ACL rupture (Lee et al., 2013b; Herzberg et al., 2017; Shagawa et al., 2021). Again, it was also determined that there was heterogeneity in such cycle-associated laxity with some individuals labeled “responders” and others “non-responders” (Schmitz and Shultz, 2013) similar to what had been shown by Park et al. (2009).

Phase of the menstrual cycle was also reported to influence the stability of the thumb, and lead to risk for degeneration of the trapeziometacarpal joint (Parker et al., 2022). The authors attributed this laxity and subsequent degeneration to serum relaxin level changes during the menstrual cycle (Parker et al., 2022). At the time of arthroplasty of this joint, it was shown that there was elevated relaxin binding activity in tissues around the joint from females but not males. In contrast, laxity of the cervical spine was reported to not to be influenced by the menstrual cycle changes (Weis et al., 2016; Parker et al., 2022).

Shultz et al. (2012) reported that serum levels for IGF-1 and markers of collagen turnover were associated with menstrual cycle-associated changes in knee laxity and potential risk for knee injury. The timing, direction of the changes and extent of the changes were influenced by whether the individuals were or were not taking oral contraceptives (Shultz et al., 2012). The authors reported that lower serum collagen production fragments and greater IGF-1 concentrations predicted greater anterior knee laxity. A limitation of such studies is that serum levels reflect total body changes in those parameters and not just those associated with the knee and therefore the correlation may not be relevant to the knee specifically. In addition, given that collagen is an essential structural component of most connective tissues of the knee (ligaments, menisci, cartilage, capsule) it may be a risky strategy to modify collagen cyclically every ∼28 days from the onset of puberty to menopause. Interestingly, in an *in vitro* generated, tissue engineered artificial ligament made with ACL cells, culturing the tissue with estrogen *in vitro* led to alterations in mechanical function without loss of collagen (Lee et al., 2015). These authors reported that exposure to estrogen led to decreases in the activity of lysyl oxidase, the enzyme that catalyzes formation of covalent collagen crosslinks. Presently, this is a correlation and details regarding how such a mechanism could work *in vivo* during multiple menstrual cycles remains to be elucidated.

An alternative hypothesis might be that the menstrual cycle-associated changes in joint laxity is regulated by changes in water content. Increases and decreases in ligament water content can influence laxity (Wallace et al., 2002). As many females appear to retain water during parts of the menstrual cycle (Bunt et al., 1989; Ramsay, 1989; Kosar et al., 2022), and skin thickness is increased in specific phases of the menstrual cycle and during pregnancy, reversible changes in water content could be responsible. The skin changes were speculated to be associated with hormone-mediated water retention (Eisenbeiss et al., 1998). In some pregnant females, pregnancy-associated changes in cornea thickness have been reported, presumably due to water retention that is reversed *postpartum* (Taradaj et al., 2018). As changes in water content would be a readily reversible strategy to influence the properties of a connective tissue, it would be preferred over one that involved the degradation and synthesis of an essential structural component such as collagen. Thus, over ∼30 years of menstrual cycles, the water content strategy would not be prone to risk for loss of the integrity of the tissues involved *via* altering the integrity of the main structural molecules, the collagens.

### 3.2 Pregnancy: Joint laxity, low back pain and risk for disease development later in life

During pregnancy many women develop joint laxity including in the spine as well as low back pain (Daneau et al., 2021; Chatprem et al., 2022; Wakkar and Pati, 2022). These complications can be at higher risk in those with hypermobility syndromes (VanderJagt and Butler, 2019; Ahlqvist et al., 2020; Pezaro et al., 2020; Robinson et al., 2022). While many women recover after pregnancy, some reports indicate that persistent post-pregnancy changes in the sacroiliac joint may contribute to the long-term postural deformities (Bailey et al., 2020) and sacroiliac joint dysfunction (Fiani et al., 2021). Sex differences in sacroiliac joint and lumbar spine degeneration have been noted and may occur *via* different process (Muellner et al., 2022). However, whether there is a subset of females whose pregnancy-associated back pain and sacroiliac involvement are causal of degenerative disease later in life remains to be confirmed by large studies.

### 3.3 Sex differences in spondyloarthritis

The term spondyloarthritis covers a heterogeneous set of conditions that can involve a variety of tissue (Sharip and Kunz, 2020) including joints and the spine, specifically the sacroiliac joints (Brown et al., 2020; Xiong et al., 2022). These inflammatory immune-mediated conditions can affect spinal tissues especially support tissues such as ligaments and their entheses of the axial skeleton (Alber et al., 2022; Li et al., 2022). Long considered mainly a disease of men, particularly ankylosing spondylitis, has more recently been reported to also affect a subset of females (Masi, 1992; Wright et al., 2020; Cunha et al., 2022; Marzo-Ortega et al., 2022; Stovall et al., 2022). Sex differences in disease presentation characteristics (Chimenti et al., 2021), as well as disease activity compared to males have been reported, with females exhibiting higher disease activity than males and exhibiting different response patterns to clinical interventions (Mease et al., 2021; Stovall et al., 2022; van der Horst-Bruinsma et al., 2022).

The onset and progression of the axial spondyloarthritis spectrum of diseases clearly has a genetic basis (Brown et al., 2020), with association with HLA B27 (Masi, 1992; Diaconu et al., 2022). Recent attempts to identify biomarkers of disease subtypes has progressed (Akhtari et al., 2020; Alber et al., 2022), as well as insights into inflammatory mechanisms (Lee et al., 2022b). In addition, recent information has revealed that the gut microbiome of patients is different from a healthy control population, but no sex differences were noted (Wang et al., 2022). Additional study of intestinal tissue and fecal samples have identified potential alterations in the tryptophan metabolizing pathways that could be involved in microbiome contributions to the disease progression (Berlinberg et al., 2021).

Thus, the sex differences in spondyloarthritis are somewhat different from many of the other diseases discussed previously in that the majority of patients are males rather than females, even though this set of diseases is believed to have an autoimmune basis. Therefore, the females with spondyloarthritis-related conditions are likely a unique subpopulation of individuals. The molecular and cellular basis for these differences has not yet been delineated, but this area is the focus of considerable investigation so hopefully, some insights will emerge in the near future to clarify these issues.

## 4 Risk for menopause-associated MSK conditions and diseases

Menopause occurs in all females at ∼45–55 years of age and the decline in menstrual cycles and sex hormone levels can be accompanied by the development of a variety of diseases or conditions in different subset of females. This area has been reviewed recently by (Hart, 2022a). An individual female does not encounter all of the post-menopausal conditions, so there is some selectivity in which are exhibited by a particular female. During most of evolutionary history the average lifespan was <40 years, so the vast majority of females did not live the make the transition to the post-menopausal state. Therefore, aside from the fundamental question of why menopause occurs at all (Lumsden and Sassarini, 2019; Hart, 2022a), questions also arise as to why these post-menopausal conditions or diseases were maintained mainly in females. One possibility is that such risks for the various post-menopausal diseases/conditions served purposes related to reproductive success. However, if that was true, why did an individual female not harbor the risks for multiple of these post-menopausal conditions. Such questions remain to be answered when more details regarding the underlying mechanisms are elucidated.

As only some of these conditions impact tissues of the MSK system, and others such as dementia and obesity affect other systems, the conditions are quite diverse (Hart, 2022a). Only those affecting the MSK system will be briefly discussed below as they are representative of conditions that can arise with the decline in sex hormones, another sex-specific transition that contributes to sex differences development of disease.

### 4.1 Osteoporosis

Osteoporosis (OP), or loss of bone integrity and increased risk for low-energy fractures (Vandenput et al., 2022), is often thought of as a disease of post-menopausal females. However, OP affects only a subset of post-menopausal females, and the ratio of females/males with OP is ∼3/1 [discussed in 21]. The treatment of males with OP is understudied (Rinonapoli et al., 2021; Vescini et al., 2021). There are also sex-differences in when fractures occur in males and females, with males suffering from fractures ∼10 years later in life than females (Vescini et al., 2021). The extent of bone loss is quite variable between individuals of either sex, so the underlying mechanisms must be complex and influenced by several factors. While there are reported sex differences in skeletal growth (Nieves, 2017), any correlations between bone growth early in life and rate of bone loss with aging and onset of menopause have not been established.

OP in females was effectively treated with hormone replacement therapy (HRT) for many years until a variety of risk factors were identified (i.e., cardiovascular disease, breast cancer, venous thromboembolism, endometrial cancer) which led to a reluctance to support such treatment (Booyens et al., 2022; Davis and Baber, 2022; Pan et al., 2022). However, this treatment option is now being revisited. In light of such reluctance regarding use of HRT, a number of other options have been developed including bisphosphonates, monoclonal antibodies against proteins involved in bone cell function, vitamin D, and anabolic peptides such as parathyroid hormone fragment and calcitonin (Zhou et al., 2014; Mulder et al., 2016; Chandran, 2022; Lim, 2022; Zhu and March 2022). Such interventions vary in effectiveness and side-effects, and thus, many patients are now using those with lower risks. However, in both men and women, OP is often the “silent disease” until a fracture occurs, so many individuals with OP are not treated, and this is especially true for men as the condition is generally viewed as a female disease. The molecular basis for why this particular subset of females is affected by OP following loss of sex hormones at menopause remains undefined. Similarly, the molecular or genetic basis for why a subset of males develop OP also remains largely unknown. Furthermore, it also remains to be determined if similar or very different mechanisms are involved. However, males do respond to bisphosphonates and other treatments (Adler, 2018; Johnston and Dagar, 2020) so there may be some commonalities.

### 4.2 Osteoarthritis

Osteoarthritis (OA) is a degenerative disease of joints such as the knee, hip, shoulder and also fingers of the hand. For many years it was presented as a disease of articular cartilage as that tissue was the one most affected. However, it is now thought of more as a disease of the whole joint, considering the joint as an organ system (Radin et al., 1991; Frank et al., 2004). As reviewed recently by Hart (Hart, 2022b), OA is now considered an inflammatory disease and there appear to be sex differences in some inflammatory markers in OA (Perruccio et al., 2019).

In the years prior to the onset of menopause, the ratio of females to males with OA is ∼1/1, but after menopause, the ratio become >2/1 [discussed in 21,22,200]. Thus, after menopause, there is an increase in the number of females with the condition compared to males. It has been proposed that this subset of post-menopausal females developing primarily knee and hip OA represent a unique and separate subtype of OA (Hart et al., 2021; Hart, 2022a; Hart, 2022b). Presently, conservative treatment options such as exercise, pain medications, and bracing are not sex specific. However, in the future some options could be sex-specific to address the potentially unique mechanisms involved in OA arising in post-menopausal females as suggested by Hart (Hart, 2022a) and Hart et al. (Hart et al., 2021). Therefore, there has been slow progress in the conservative treatment realm for OA, and for many patients this leads to a total joint replacement for the condition.

Thus, in a subset of post-menopausal females the loss of sex hormones leads to alterations in joint regulatory mechanisms leading to OA. What is uniquely different in this subset and how sex hormone loss contributes to the OA development, remains to be elucidated for the most part.

### 4.3 Sarcopenia

Sarcopenia is the age-related loss of muscle structure and function (i.e., muscle mass and strength) and can occur in both males and females, but there are some sex-associated differences in the onset and progression of such muscle loss (Du et al., 2019; Buckinx and Aubertin-Leheudre, 2022; McMillian et al., 2022). Sarcopenia can also be influenced by associated obesity in some patients (Jia et al., 2022; Muollo et al., 2022). Sarcopenia and sarcopenic obesity in older individuals can influence other age- and sex-related conditions such as osteoporosis (Edwards et al., 2015), cardiovascular fitness (Billingsley et al., 2022), osteoarthritis (Godziuk et al., 2020), and cognitive performance (Cavazzotto et al., 2022), all affecting different subsets of post-menopausal females compared to age-matched males.

Therefore, sarcopenia as an age-related loss of muscle integrity would appear to interface with other post-menopause conditions to impact disease activity and progression. As some post-menopausal females also gain weight after menopause, likely due to loss of estrogen’s effect on energy balance (De Jesus and Henry, 2022; Mahboobfard et al., 2022), sarcopenic obesity is also very prevalent in this population, a finding that may enhance the impact of the other conditions discussed above.

### 4.4 Intravertebral disc degeneration

Estrogen is believed to play a role in intravertebral disc (IVD) degeneration (Wang and Griffith, 2010; Jin et al., 2020). In the post-menopausal state, the presenting symptom is usually pain (Wang, 2016). The IVD degeneration types include adult-onset lumbar scoliosis (Aebi, 2005; Rumancik et al., 2005; Urrutia et al., 2011a; Urrutia et al., 2011b) or spondylolisthesis (Jacobsen et al., 2007; Cholewicki et al., 2017). The causes for the development of such conditions in post-menopausal women is mainly unknown, but some associations with parity have been made (Cevik et al., 2020), and risk factors for pregnancy-related pelvic girdle pain investigated (Wuytack et al., 2020), as well as a focus on pregnancy-associated changes in motor control of the spine (Desgagnes et al., 2022). Some evidence that exercise can help prevent development of low back pain has appeared, but it is not compelling as yet (Santos et al., 2022). In addition, some evidence for the role of HRT in preventing developing of some post-menopausal spondylolisthesis has been generated (Marty-Poumarat et al., 2012), potentially implicating the loss of estrogen in develop of the condition rather than a consequence of pregnancy-associated factors alone.

In summary, a number of MSK conditions arise in subsets of post-menopausal females at rates higher than age-matched males, or associated with conditions uniquely associated with females (i.e., pregnancy). Some of these can be linked more directly to loss of sex hormones than others, but clearly there are significant sex differences that have been noted. Of interest is the differences between adolescent idiopathic scoliosis which appears to arise in the thoracic spine post-puberty and onset of increased levels of sex hormones, and adult scoliosis which is associated with the lumbar spine and arises in the post-menopausal state following loss of sex hormones. The development of adult scoliosis can be a “re-activation” of somewhat quiescent AIF or not but does appear to occur rapidly in the post-menopausal state in a subset of patients (Urrutia et al., 2011a; Marty-Poumarat et al., 2012). Why it occurs in a particular subset is currently unknown. In a recent Clinical Case Series with 187 patients having Adult Symptomatic Lumbar Scoliosis (ASLS), >90% were female (Carreon et al., 2020). Therefore, there is a majority of patients with ASLS that are post-menopausal females (mean age >58 years) (Carreon et al., 2020). Details regarding the characteristics of the males with ASLS could not be found.

At the surface, these differences may be difficult to reconcile, but it is also clear that many epigenetic changes occur at the time of puberty (Hart, 2022a) and also from life experiences, so the loss of sex hormones following menopause likely does not return females to the pre-puberty state. Thus, the basis for the development of post-menopausal conditions is likely complex and multifactorial.

## 5 Analysis of sex differences in risk for MSK conditions and diseases

It is clear from the above discussions that sex differences exist for a variety of MSK conditions across the lifespan, from development into the post-menopausal years. As some of these conditions or diseases occur prior to the onset of puberty, others after the onset of puberty, and then again others after menopause, the fundamental causes of the conditions cannot be related directly to sex hormone levels. Certainly, some hormonal changes associated with puberty, menstrual cycles, and pregnancy have been correlated with risk for joint injuries or disease, but it must be remembered that only a small percent of females in each “category” are affected. Thus, other variables such as genetic factors or epigenetic alterations are likely also involved in the risk.

Some of the response patterns associated with sex differences may be imprinted during *in utero* development when hormones from the mother as well as locally produced hormones set the stage for future hormone-related events associated with puberty and menopause in females contribute to the elaboration of some of the risks for loss of MSK tissue integrity.

The presence of sex hormone receptors in most tissues is relatively constant across the lifespan, although levels and functioning for some receptors can vary across the menstrual cycle (Zelenko et al., 2012; Kruger et al., 2023) and during aging (Gardini et al., 2020; Oveisgharan et al., 2023). Some of such changes can be cell and tissue-specific and are potentially due to methylation of the receptor genes (Penolazzi et al., 2004; Tsuboi et al., 2017). Cells from tissues of both males and females express sex hormone receptors (Liu et al., 1996; Sciore et al., 1998; Yu et al., 2001) and both sexes make and respond to both estrogens and testosterone (Hammes and Levin, 2019; Ipulan-Colet, 2022). Some tissues may express different levels of estrogen receptors (ER-alpha, ER-beta, plasma membrane-associated variants) and androgen receptors (Petersen et al., 1998; Pujol et al., 1998; Walters and Nemere, 2004; Song and Santen, 2006; Chang et al., 2013; Madeira et al., 2013). Studies from the author’s laboratory have indicated that estrogen receptors can exert effects on the expression of proteinase genes *via* interactions with the promoter region in model systems (Lu et al., 2006; Lu et al., 2006; Achari et al., 2008; Achari et al., 2009; Thaler et al., 2014). Such activities by ER were dampened by the addition of their hormone ligand estrogen. Furthermore, the activity of ER-beta in this regard was greater than that for ER-alpha. Thus, in the absence of hormone, ER could still exert effects on cells and potentially account for some of the post-menopausal conditions arising after menopause in subsets of females. Whether the potential effects of the ER could be affected by anabolic growth regulators in the pre-puberty phase of the lifespan remains to be determined. It also remains to be determined whether similar activities are associated with androgen receptors and those for progesterone.

The findings and speculations that muscle imbalances in the femur and in the spine of post-puberty females may be involved in the risk for ACL injuries and adolescent idiopathic scoliosis, respectively, is of interest. Why and how such imbalances develop, and their bilaterality would implicate that they develop in a subset of females during development but then the differences are elaborated following the onset of puberty and initiation of growth leading to maturation. In the case of AIS, the variation in the extent of the curvature may mean that either other factors in addition to such muscle influences are involved, or such variation resides within the muscles themselves and the variation arises due to variation in the hormone-hormone receptor consequences in the muscle tissue.

In the case of AIS, the imbalances would have a direct effect on the regulation of growth of the spine tissue. However, the femoral muscle imbalances may never be detected or matter if the individual did not participate in sports that depend on a balance between the quadriceps and hamstring muscles to prevent non-contact injuries to components of the knee such as the ACL. While not evident from the literature, it would be of interest to determine whether the occurrence of muscle imbalances in the femur occurred in the same subpopulation that experiences AIS. In addition, it remains unclear whether the basis for the muscle imbalances in AIS and the femur arise during development and are just elaborated after onset of puberty, or whether it arises after puberty *via* some as yet unknown differential effect of sex hormones on specific muscle growth and maturation.

While many aspects of sex-specific biological differences appear to be “imprinted” during fetal development and thus onset of puberty merely enhances an established blueprint, it is also possible that epigenetic events arising during early post-natal growth and maturation could also play a more individualized role in the response pattern to the mediators arising with the onset of puberty that are responsible for further growth and conditions leading to sexual maturation in a variety of relevant systems including the MSK system (Chmielewska et al., 2020; Shepherd et al., 2021; Szyf, 2021; Hart, 2022a; Gegenhuber and Tolkuhn, 2022; Monotas et al., 2022). As sex hormones can both modify the activity of sex hormone receptors (Lu et al., 2006; Lu et al., 2006; Achari et al., 2008; Achari et al., 2009; Thaler et al., 2014), as well as modify other gene expression patterns as sex hormone-receptor complexes (Fuentes and Silveya, 2019; Kovacs et al., 2020; Mayayo-Peralta et al., 2021), the molecular basis for sex differences in the regulation of MSK tissues across the lifespan can take many forms. Clearly, as puberty leads to epigenetic alterations, and then further epigenetic alterations can occur in females associated with pregnancies, the loss of cycling hormone levels following onset of menopause is not a return to the pre-puberty state. In males, life experiences and environmental exposures could also potentially lead to epigenetic alterations that could influence age-related MSK conditions and diseases.

Based on the above discussion some of the sex differences regarding the regulation of MSK tissues may reside in differences in neuroregulation. All MSK tissues except for articular cartilage are innervated to some degree, innervation that is associated with proprioception and regulation of the integrated functioning of the tissues for mobility and environmental navigation (Ackermann et al., 2016; Hart, 2018), as well as providing input into different tissues cellular and molecular responses (Murphy and Hart, 1993; Hart and Reno, 1998; Hart et al., 1999; Salo et al., 2007; Scott et al., 2007; Beye et al., 2008; Bring et al., 2012; Ackermann et al., 2014). Sex differences in brain maturation can occur in childhood and adolescence (De Bellis et al., 2001; Campbell et al., 2005; Abel and Rissman, 2012; Koolschijn and Crone, 2013) and thus potentially contribute to sex-specific differences in MSK tissue risks and regulation. In addition to direct effects of neural regulation on tissues of the MSL system, neural regulation of the vascular components of such tissues can also occur in a sex-dependent manner (Joyner et al., 2015; Charkoudian et al., 2017; DeLorey, 2021; Klassen et al., 2021). Such sex differences may play a role in adapted vasoregulation during pregnancy (McDougall et al., 1998; McDougall et al., 2000). Therefore, sex-differences in the regulation of MSK tissues may be regulated at multiple levels, with some directly influenced by sex hormones, but others indirectly *via* sex-differences in vasoregulation and neuroregulation.

Some insights regarding the complexity of sex-specific regulation of the MSK system comes from the study of muscles. Muscles, like all MSK tissues, are regulated by mechanical loading and subscribe to the “use it or loss it” principle (Hart, 2021). Therefore, exercising can lead to growth of muscles with adaptation to a new functional level. Such exercising and loading of bone can also lead to enhanced bone qualities (Hart, 2021). Disuse can lead to atrophy of both muscles and bone, such as in space flight [discussed in 276]. As discussed earlier, use of neuromuscular training programs can overcome the risk for ACL tears in female athletes with muscle imbalances in the femur but if the exercises are not continued, the risk returns to an elevated state (Myklebust et al., 2013), presumably due to a return to the imbalanced state. Thus, the “set point” for the bilateral muscle imbalances may be defined early in life, and while adaptive, it returns to a state that was likely defined during growth and maturation. However, this hypothesis remains to be proven by additional research. Since the muscle imbalances are bilateral in nature, the neuroregulation of the tissue likely defines the muscle characteristics arise during development, early growth and maturation, adolescence and then maintained at skeletal maturity. Thus, even in the presence of changing sex hormone levels during the menstrual cycle, the muscle imbalances are maintained. The menstrual cycle can also impact the peripheral vascular function in pre-menopausal females (Williams et al., 2020) and exercise metabolism (Oosthuyse and Bosch, 2010), so the imbalance can still be maintained when multiple variables could potentially exert an influence on muscle function. While not yet proven, these muscle imbalances could likely arise *via* regulatory imprinting during development and growth and maturation, as well as additional factors such as to further epigenetic alterations to the regulation of the affected tissues (Gegenhuber and Tolkuhn, 2022; Monotas et al., 2022). In the knee joint, laxity is overtly affected by the menstrual cycle in ∼80% of young females (Park et al., 2009), but in 20% the individuals are resistant to changes associated with fluctuating sex hormone levels indicating they may be directly regulated independent of the hormones such as estrogen. For the 80% of females who were affected, the changes may be associations and not direct “cause and effect’ relationships. To better understand such relationships, further research will be required.

The above discussion also raises the interesting speculation that perhaps the myriad of conditions that can affect subsets of post-menopausal females, as well as potentially other sex-specific risk factors for injury and disease have at least some of the mechanistic basis an indirect effect *via* neuroregulation of the microvascular systems in specific target tissues, as well as variation in direct neuroregulation of specific target tissues. As the functionality of specific MSK tissues requires integration of the target cells in each tissue and the mechanical environment + the microvasculature + the innervation, any potential “defects” in such integration could lead to diseases or conditions developing at different stages of the lifespan, a lifespan that likely have more transitions for females than males. Such conceptual thinking could provide some insights into commonalities for the various conditions/diseases discussed, particularly those arising in the postmenopausal state. Thus, rather than completely focusing on the target tissues in isolation, perhaps a different approach could lead to improved understanding of the mechanisms involved.

Future studies should further investigate the different potential regulatory features of tissues of the MSK system to assess the relative contributions of direct effects of sex hormones, and potential indirect neurovascular mechanisms as well as direct effects of neuro elements on the target tissues. Thus, some tissue changes are likely associated with the menstrual cycle and accompanying hormone level changes, but “cause and effect” may not be direct in the regulation of the tissues and their risk for injury and disease.

## 6 Conclusion

Sex differences in risk for diseases or injury involving tissues of the MSK system exist across the lifespan. While some of the risk may be associated with sex-specific events (i.e., pregnancy), still other sex-dependent risks appear to be associated with growth and maturation, puberty, and menopause. Thus, these risks can become evident prior to onset of increases in sex hormones, following the onset of increases in such hormones at puberty, and then later in life when sex hormones decrease associated with menopause. This latter stage of life is also interfaced with aging variables.

Thus, while there are sex differences in risk for MSK tissue injury or diseases, it is not absolute and both sexes can be affected but to differing extent. In the case of sex differences in ACL rupture risk, the risk can be lowered to ∼1/1 *via* neuromuscular training programs so the sex differences can be modified. For other conditions such OP, prevention of fracture risk can be diminished with hormone replacement or anti-resorption drugs in females, and with anti-resorption drugs in males. Therefore, diminishing risk can be accomplished for many of these patients but this approach does not address the fundamental mechanisms causing these conditions.

From the above discussion, it is clear that the risk for different types of injuries or diseases involving MSK tissues, particularly for females, varies from prior to puberty, post-puberty, and then in the post-menopausal state. Therefore, it remains unclear if (and how) sex hormones are directly contributing to the observed sex differences by causing the conditions! As many of the templates for regulation of MSK tissues are laid down during development, it may be that the sex-hormone environment affects the fetus with development of a genetic/epigenetic “blueprint” to set the stage for the elaboration of risks later in life. Clearly, neonatal hip dysplasia arises during development and is evident at the time of birth and is framed by the *in-utero* environment. Thus, sex hormones may contribute to the elaboration of the post-natal risks that are sex-dependent *via* modulation of pre-existing risk arising during development.

For individual patients or potential patients, prevention of disease or injury is a paramount importance and understanding the molecular and genetic basis for the risk is critical for the individual. Therefore, better understanding of the scientific basis for the risks discussed should lead to development of improved treatments, prevention strategies, and identification of those subsets of females and males with specific risks. Certainly, human heterogeneity (i.e., genetics, epigenetics, environmental variables) can contribute to the complexity of such understanding and the deciphering of the basis for such risks may be complex with “one solution” not fitting all individuals.

## References

[B1] Abel J. L. Rissman E. F. (2012). Location, location, location: Genetic regulation of neural sex differences. Rev. Endocr. Metab. Disord. 13, 151–161. 10.1007/s11154-011-9186-0 21607612 PMC3622700

[B2] Achari Y. Lu T. Hart D. A. (2008). Polymorphisms in the promoter regions for human MMP-1 and MMP-13 lead to differential responses to the alpha and beta isoforms of estrogen receptor and their ligand *in vitro* . Biochim. Biophys. Acta 1782, 391–400. 10.1016/j.bbadis.2008.02.009 18358246

[B3] Achari Y. Lu T. Katzenellenbogen B. S. Hart D. A. (2009). Distinct roles for AF-1 and -2 of ER-alpha in regulation of MMP-13 promoter activity. Biochim. Biophys. Acta 1792, 211–220. 10.1016/j.bbadis.2009.01.002 19185056

[B4] Ackermann P. W. Franklin S. L. Dean B. J. F. Carr A. J. Salo P. T. Hart D. A. (2014). Neuronal pathways in tendon healing and tendinopathy-update. Front. Biosci. (Landmark Ed. 19, 1251–1278. 10.2741/4280 24896349

[B5] Ackermann P. W. Salo P. Hart D. A. (2016). Tendon innervation. Adv. Exp. Med. Biol. 920, 35–51. 10.1007/978-3-319-33943-6_4 27535247

[B6] Adler R. A. (2018). Update on osteoporosis in men. Best. Pract. Res. Clin. Endocrinol. Metab. 32, 759–772. 10.1016/j.beem.2018.05.007 30449553

[B7] Aebi M. (2005). The adult scoliosis. Eur. Spine J. 14, 925–948. 10.1007/s00586-005-1053-9 16328223

[B8] Ahlqvist K. Bjelland E. K. Pingel R. Schlager A. Nilsson-Wikmar L. Kristiansson P. (2020). The association of self-reported generalized joint hypermobility with pelvic girdle pain during pregnancy: A retrospective cohort study. BMC Musculoskelet. Disord. 21, 474. 10.1186/s12891-020-03486-w 32689990 PMC7372850

[B9] Ahmed S. Ali S. R. Ishaque S. Sami N. (2014). Clinical and biochemical characteristics of children with juvenile idiopathic arthritis. J. Coll. Physicians Surg. Pak 24, 498–502. 25052974

[B10] Akhtari M. Zargar S. J. Vojdanian M. Ashraf-Ganjoueli A. Javinani A. Hamzeh E. (2020). P2 receptors mRNA expression profiles in macrophages from ankylosing spondylitis patients and healthy individuals. Int. J. Rheum. Dis. 23, 350–357. 10.1111/1756-185X.13783 31884692

[B11] Alber S. Kumar S. Liu J. huang Z. M. Paez D. Hong J. (2022). Single cell transcriptome and surface epitope analysis of ankylosing spondylitis facilitates disease classification by machine learning. Front. Immunol. 13, 838636. 10.3389/fimmu.2022.838636 35634297 PMC9135966

[B12] Angeles-Han S. T. Pelajo C. F. Vogler L. B. Rouster-Stevens K. Kennedy C. Ponder L. (2013). Risk markers of juvenile idiopathic arthritis-associated uveitis in the Childhood Arthritis and Rheumatology Research Alliance (CARRA) registry. J. Rheumatol. 40, 2088–2096. 10.3899/jrheum.130302 24187099 PMC4117408

[B13] Arhos E. K. Di Stasi S. Hartigan E. H. Synder-Mackler L. (2022). Males and females have different muscle activity patterns during gait after ACL injury and reconstruction. J. Electromyogr. Kinesiol 66, 102694. 10.1016/j.jelekin.2022.102694 35988533 PMC9588796

[B14] Ayanoglu S. Cabuk H. Cabuk F. K. Beng K. Yildirim T. Bozkurt S. U. (2021). Greater presence of receptors for relaxin in the ligamentum teres of female infants who under go open reduction for developmental dysplasia of the hip. J. Orthop. Surg. Res. 16, 627. 10.1186/s13018-021-02784-w 34663407 PMC8524823

[B15] Bailey J. F. Sparrey C. J. Williams F. M. K. Curran P. F. Lotz J. C. Kramer P. A. (2020). The effect of parity on age-related degenerative changes in sagittal balance. Spine (Phila Pa 1776 45, E210–E216. 10.1097/BRS.0000000000003234 31513113

[B16] Barber-Westin S. D. Noyes F. R. Smith S. T. Campbell T. M. (2009). Reducing the risk of noncontact anterior cruciate ligament injuries in the female athlete. Phys. Sportsmed. 37, 49–61. 10.3810/psm.2009.10.1729 20048528

[B17] Barnes M. G. Grom A. A. Thompson S. D. Griffin T. A. Luyrink L. K. Clbert R. A. (2010). Biologic similarities based on age at onset in oligoarticular and polyarticular subtypes of juvenile idiopathic arthritis. Arthritis Rheum. 62, 3249–3258. 10.1002/art.27657 20662067 PMC3018072

[B18] Barrera C. A. Cohen S. A. Sankar W. N. Ho-Fung V. M. Sze R. W. Nguyen J. C. (2019). Imaging of developmental dysplasia of the hip: Ultrasound, radiography and magnetic resonance imaging. Pediatr. Radiol. 49, 1652–1668. 10.1007/s00247-019-04504-3 31686171

[B19] Berlinberg A. J. Regner E. H. Stahly A. Brar A. Reisz J. A. Gerich M. E. (2021). Multi’omics analysis of intestinal tissue in ankylosing spondylitis identifies alterations in the tryptophan metabolism pathway. Front. Immunol. 12, 587119. 10.3389/fimmu.2021.587119 33746944 PMC7966505

[B20] Berntson L. Damgard M. Andersson-Gare B. Herlin T. Nielson S. Nordal E. (2008). HLA-B27 predicts a more extended disease with increasing age at onset in boys with juvenile idiopathic arthritis. J. Rheumatol. 35, 2055–2061. 18785306

[B21] Betz R. R. Bunnell W. P. Lambrecht-Muller E. MacEwen G. D. (1987). Scoliosis and pregnancy. J. Bone Jt. Surg. Am. 69, 90–96. 10.2106/00004623-198769010-00015 2948962

[B22] Beye J. A. Hart D. A. Bray R. C. Seerattan R. A. McDougall J. J. Leonard C. A. (2008). Denervation alters mRNA levels of repair-associated genes in a rabbit medial collateral ligament injury model. J. Orthop. Res. 24, 1842–1853. 10.1002/jor.20219 16865716

[B23] Billingsley H. E. Del Buono M. G. Canada J. M. Kim Y. Damonte J. I. Trankle C. R. (2022). Sarcopenic obesity is associated with reduced cardiorespiratory fitness compared with nonsarcopenic obesity in patients with heart failure with reduced ejection fraction. Circ. heart Fail 15, e009518. 10.1161/CIRCHEARTFAILURE.122.009518 36098058 PMC9588574

[B24] Black A. L. Clark A. L. (2022). Sexual dimorphism in knee osteoarthritis: Biomechanical variances and biological influences. J. Orthop. Res. 32, 104–108. 10.1016/j.jor.2022.05.016 PMC9166391 35668833

[B25] Booyens R. M. Engelbrecht A. M. Strauss L. Pretorius E. (2022). To clot, or not to clot: The dilemma of hormone treatment options for menopause. Thromb. Res. 218, 99–111. 10.1016/j.thromres.2022.08.016 36030662

[B26] Bowden D. Michielli A. Merrill M. Will S. (2022). Systematic review and meta-analysis for the impact of rod materials and sizes in the surgical treatment of adolescent idiopathic scoliosis. Spine Deform. 10, 1245–1263. 10.1007/s43390-022-00537-1 35737287 PMC9579082

[B27] Bring D. K. Paulson K. P. Renstrom P. Salo P. Hart D. A. Ackermann P. W. (2012). Residual substance P levels after capsaicin treatment correlate with tendon repair. Wound Repair Regen. 20, 50–60. 10.1111/j.1524-475X.2011.00755.x 22276586

[B28] Brophy R. H. Silvers H. J. Mandelbaum B. R. (2010). Anterior cruciate ligament injuries: Etiology and prevention. Sports Med. Arthrosc. Rev. 18, 2–11. 10.1097/JSA.0b013e3181cdd195 20160623

[B29] Brown M. A. Xu H. Li Z. (2020). Genetics and the axial spondyloarthritis spectrum. Rheumatol. Oxf. 59, iv58–iv66. 10.1093/rheumatology/keaa464 PMC7566537 33053195

[B30] Brunello F. Tirelli F. Pegoranro L. Dell’Apa F. Alfisi A. Calzamatta G. (2022). New insights on juvenile psoriatic arthritis. Front. Pediatr. 10, 884727. 10.3389/fped.2022.884727 35722498 PMC9199423

[B31] Buchan J. G. Alvarado D. M. Haller G. E. Cruchaga C. Harms M. B. Zhang T. (2014). Rare variants in FBN1 and FBN2 are associated with severe adolescent idiopathic scoliosis. Hum. Mol. Genet. 23, 5271–5282. 10.1093/hmg/ddu224 24833718 PMC4159151

[B32] Buckinx F. Aubertin-Leheudre M. (2022). Sarcopenia in menopausal women: Current perspectives. Int. J. Women’s Health 14, 805–819. 10.2147/IJWH.S340537 35769543 PMC9235827

[B33] Bunt J. C. Lohman T. G. Boileau R. A. (1989). Impact of total body water fluctuations on estimation of body fat from body density. Med. Sci. Sports Exerc 21, 96–100. 10.1249/00005768-198902000-00017 2927308

[B34] Burger M. Coetzee W. du Plessis L. Z. Geldenhuys L. Joubert F. Myburgh E. (2019). The effectiveness of schroth exercises in adolescents with idiopathic scoliosis: A systematic review and meta-analysis. S Afr. J. Physiother. 75, 904. 10.4102/sajp.v75i1.904 31206094 PMC6556933

[B35] Burwell R. G. Clark E. M. Dangerfield P. H. Moulton A. (2016). Adolescent idiopathic scoliosis (AIS): A multifactorial cascade concept for pathogenesis and embryonic origin. Scoliosis Spinal Disord. 11, 8. 10.1186/s13013-016-0063-1 27252984 PMC4888516

[B36] Butler L. S. Janosky J. J. Sugimoto D. (2022). Pediatric and adolescent knee injuries: Risk factors and preventive strategies. Clin. Sports Med. 41, 799–820. 10.1016/j.csm.2022.05.011 36210172

[B37] Campbell I. G. Darchia N. Khaw W. Y. Higgins L. M. Feinberg I. (2005). Sleep EEG evidence of sex differences in adolescent brain maturation. Sleep 28, 637–643. 10.1093/sleep/28.5.637 16171278 PMC2596759

[B38] Carreon L. Y. Glassman S. D. Yanik E. L. Kelly M. P. Lurie J. D. Bridwell K. H. (2020). Differences in functional treadmill tests in patients with adult symptomatic lumbar scoliosis treated operatively and nonoperatively. Spine 45, E1476–E1482. 10.1097/BRS.0000000000003640 33122605

[B39] Cattalini M. Soliani M. Caparello M. C. Cimaz R. (2019). Sex differences in pediatric rheumatology. Clin. Rev. Allergy Immunol. 56, 293–307. 10.1007/s12016-017-8642-3 28849549

[B40] Cavazzotto T. G. de Campos C. D. V. Mazur C. E. da Silva D. F. Valero J. M. S. Vieira E. R. (2022). Association between cognitive performance and sarcopenic obesity in older adults with Alzheimer’s disease. Dement. Neuropsychol. 16, 28–32. 10.1590/1980-5764-DN-2021-0039 35719255 PMC9170256

[B41] Cevik S. Yilmaz H. Kaplan A. Yetkinel S. Evran S. Calis F. (2020). Association between parity and lumbar spine degenerative disorders in young women. Br. J. Neurosurg. 34, 172–175. 10.1080/02688697.2019.1701628 31851846

[B42] Chandran M. (2022). The why and how of sequential and combination therapy in osteoporosis. A review of the current evidence. Arch. Endocrinol. Metab. 66, 724–738. 10.20945/2359-3997000000564 36382762 PMC10118820

[B43] Chang C. Yeh S. Lee S. O. Chang T. M. (2013). Androgen receptor (AR) pathophysiological roles in androgen-related diseases in skin, bone/muscle, metabolic syndrome and neuron/immune systems: Lessons learned from mice lacking AR in specific cells. Nucl. Recept Signal 11, e001. 10.1621/nrs11001 24653668 PMC3960937

[B44] Charkoudian N. Hart E. C. J. Barnes J. N. Joyner M. J. (2017). Autonomic control of body temperature and blood pressure: Influences of female sex hormones. Clin. Auton. Res. 27, 149–155. 10.1007/s10286-017-0420-z 28488202

[B45] Chatprem T. Puntumetakul R. Siritaratiwat W. Hunsawong T. Boucaut R. (2022). Prevalence of Thai people with lumbar instability and associated factors: A cross-sectional study. J. Pain Res. 15, 3287–3297. 10.2147/JPR.S381270 36304488 PMC9592730

[B46] Cheung J. P. Y. Cheung P. W. H. Luk K. D. K. (2019). When should we wean bracing for adolescent idiopathic scoliosis? Clin. Orthop. Relat. Res. 477, 2145–2157. 10.1097/CORR.0000000000000781 31135558 PMC7000074

[B47] Chiaroni-Clarke R. C. Munro J. E. Ellis J. A. (2016). Sex bias in paediatric autoimmune disease-not just about sex hormones? J. Autoimmun. 69, 12–23. 10.1016/j.jaut.2016.02.011 26970680

[B48] Chimenti M. S. Alten R. D’Agnostino A. A. Gremese E. Kiltz U. Lubrano E. (2021). Sex-associated and gender-associated differences in the diagnosis and management of axial spondyloarthritis: Addressing the unmet needs of female patients. RMD Open 7, e001681. 10.1136/rmdopen-2021-001681 34876490 PMC8655606

[B49] Chmielewska M. Janusz P. Andrusiewicz M. Kotwicke T. Kotwicke M. (2020). Methylation of estrogen receptor 2 (ESR2) in deep paravertebral muscles and its association with idiopathic scoliosis. Sci. Rep. 10, 22331. 10.1038/s41598-020-78454-4 33339862 PMC7749113

[B50] Cholewicki J. Lee A. S. Popovich J. M. Mysliwiec L. W. Winkelpleck M. D. Flood J. N. (2017). Degenerative spondylolisthesis is related to multiparity and hysterectomies in older women. Spine (Phila Pa 1776) 42, 1643–1647. 10.1097/BRS.0000000000002178 PMC5623604 28368984

[B51] Chung L. Y. Nam H. K. Rhie Y. J. Huh R. Lee K. H. (2020). Prevalence of idiopathic scoliosis in girls with central precocious puberty: Effect of a gonadotropin-releasing hormone agonist. Ann. Pediatr. Endocrinol. Metab. 25, 92–96. 10.6065/apem.1938164.082 32615688 PMC7336263

[B52] Cunha R. N. Vieira-Sousa E. Khmelinski N. Avila-Ribeiro P. Couto M. Seixas M. I. (2022). Sex differences in axial spondyloarthritis: Data from a Portuguese spondyloarthritis cohort. ARP Rheumatol. 1, 42–48. 35633576

[B53] Daneau C. Abboud J. Marchand A. A. Houle M. Pasquier M. Ruchat S. M. (2021). Mechanisms underlying lumbopelvic pain during pregnancy: A proposed model. Front. Pain Res. (Lausanne) 2, 773988. 10.3389/fpain.2021.773988 35295430 PMC8915559

[B54] Davis S. R. Baber R. J. (2022). Treating menopause-MHT and beyond. Nat. Rev. Endocrinol. 18, 490–502. 10.1038/s41574-022-00685-4 35624141

[B55] Day J. M. Fletcher J. Goghlan M. Ravine T. (2019). Review of scoliosis-specific exercise methods used to correct adolescent idiopathic scoliosis. Arch. Physiother. 9, 8. 10.1186/s40945-019-0060-9 31463082 PMC6708126

[B56] De Azevedo G. B. L. Perini J. A. AraujoMoliterno A. E. P. L. A. M. jr Andrande R. M. Guimaraes J. A. M. Defino H. L. A. (2022). Association of FBN1 polymorphism with susceptibility of adolescent idiopathic scoliosis: A case-control study. BMC Musculoskelet. Disord. 23, 430. 10.1186/s12891-022-05370-1 35526034 PMC9077855

[B57] De Bellis M. D. Keshavan M. S. Beers S. R. Hall J. Frustaci K. Masalehdan A. (2001). Sex differences in brain maturation during childhood and adolescence. Cereb. Cortex 11, 552–557. 10.1093/cercor/11.6.552 11375916

[B58] de Hundt M. Vlemmix F. Bais J. M. Hutton E. K. de Groot C. J. Mol B. W. J. (2012). Risk factors for developmental dysplasia of the hip: A meta-analysis. Eur. J. Obstet. Gynecol. Reprod. Biol. 165, 8–17. 10.1016/j.ejogrb.2012.06.030 22824571

[B59] De Jesus A. N. Henry B. A. (2022). The role of oestrogen in determining sexual dimorphism in energy balance. J. Physiol. 601, 435–449. 10.1113/JP279501 36117117 PMC10092637

[B60] de Oliveira Barbosa R. Albernaz E. P. (2019). Profile of patients diagnosed with developmental dysplasia of the hip. Rev. Bras. Ortop. San. Paulo) 54, 497–502. 10.1016/j.rbo.2018.02.005 PMC6855922 31736518

[B61] De Salvatore S. Ruzzini L. Longo U. G. Marino M. Greco A. Piergentili I. (2022). Exploring the association between specific genes and the onset of idiopathic scoliosis: A systematic review. BMC Med. Genomics 15, 115. 10.1186/s12920-022-01272-2 35590413 PMC9118580

[B62] DeLorey D. S. (2021). Sympathetic vasoconstriction in skeletal muscle: Modulatory effects of aging, exercise training, and sex. Appl. Physiol. Nutr. Metab. 46, 1437–1447. 10.1139/apnm-2021-0399 34348066

[B63] Desgagnes A. Patricio P. Berube N. Bernard S. Lamothe M. Masse-Alarie H. (2022). Motor control of the spine in pregnancy-related lumbopelvic pain: A systematic review. Clin. Biomech. (Bristol Avon) 88, 105716. 10.1016/j.clinbiomech.2022.105716 35843136

[B64] Desteli E. E. Piskin A. Gulman A. B. Kaymaz F. Koksal B. Erdogan M. (2013). Estrogen receptors in hip joint capsule and ligamentum capitis femoris of babies with developmental dysplasia of the hip. Acta Orthop. Traumatol. Turc 47, 158–161. 10.3944/aott.2013.2772 23748614

[B65] Dewan M. C. Mummareddy N. Bonfield C. (2018). The influence of pregnancy on women with adolescent idiopathic scoliosis. Eur. Spine J. 27, 253–263. 10.1007/s00586-017-5203-7 28664223

[B66] Di Nardo F. Laureati G. Strazza A. Memgarelli A. Burattini L. Agnostini V. (2017). Is child walking conditioned by gender: Surface EMG patterns in female and male children. Gait Posture 53, 254–259. 10.1016/j.gaitpost.2017.02.009 28236775

[B67] Diaconu A. D. Ceasovschih A. Sorodoc V. Pomirleanu C. Lionte C. Sorodor L. (2022). Practical significance of biomarkers in axial spondyloarthritis: Updates on diagnosis, disease activity, and prognosis. Int. J. Mol. Sci. 23, 11561. 10.3390/ijms231911561 36232862 PMC9570274

[B68] Diarbakerti E. Grauers A. Danielson A. Gerdhem P. (2018). Health-related quality of life in adulthood in untreated and treated individuals with adolescent or juvenile idiopathic scoliosis. J. Bone Jt. Surg. Am. 100, 811–817. 10.2106/JBJS.17.00822 29762275

[B69] Du Y. Wang X. Xie H. Zheng S. Wu X. Zhu X. (2019). Sex differences in the prevalence and adverse outcomes of sarcopenia and sarcopenic obesity in community dwelling elderly in East China using the AWGS criteria. BMC Endocr. Disord. 19, 109. 10.1186/s12902-019-0432-x 31653213 PMC6814981

[B70] Dufvenberg M. Diabakerli E. Charalampidis A. Oberg B. Tropp H. Ahl A. A. (2021). Six-month results on treatment adherence, physical activity, spinal appearance, spinal deformity, and quality of life in an ongoing randomised trial on conservative treatment for adolescent idiopathic scoliosis (CONTRAIS). J. Clin. Med. 10, 4967. 10.3390/jcm10214967 34768487 PMC8585057

[B71] Edison B. R. Pandya N. Ratel N. M. Carter C. W. (2022). Sex and gender differences in pediatric knee injuries. Clin. Sports Med. 42, 769–787. 10.1016/j.csm.2022.06.002 36210170

[B72] Edwards M. H. Dennison E. M. Sayer A. A. Fielding R. Cooper C. (2015). Osteoporosis and sarcopenia in older age. Bone 80, 126–130. 10.1016/j.bone.2015.04.016 25886902 PMC4601530

[B73] Eisenbeiss C. Welzel J. Schmeller W. (1998). The influence of female sex hormones on skin thickness: Evaluation using 20 MHz sonography. Br. J. Dermatol 139, 462–467. 10.1046/j.1365-2133.1998.02410.x 9767291

[B74] Ellison T. M. Flagstaff I. Johnson A. E. (2021). Sexual dimorphisms in anterior cruciate ligament injury: A current concepts review. Orthop. J. Sports Med. 9, 23259671211025304. 10.1177/23259671211025304 34993256 PMC8725014

[B75] Emery C. A. Roy T. O. Whittaker J. L. Nettel-Aguirre A. van Mechelan W. (2015). Neuromuscular training injury prevention strategies in youth sport: A systematic review and meta-analysis. Br. J. Sports Med. 49, 865–870. 10.1136/bjsports-2015-094639 26084526

[B76] Eng S. W. M. Aeschlimann F. A. van Veenedaal M. Berard R. A. Rosenberg A. M. Morris Q. (2019). Patterns of joint involvement in juvenile idiopathic arthritis and prediction of disease course: A prospective study with multilayer non-negative matrix factorization. PLoS Med. 16, e1002750. 10.1371/journal.pmed.1002750 30807586 PMC6390994

[B77] Esposito T. Uccello R. Caliendo R. De Martino G. F. Carnevale U. A. G. Cuomo S. (2009). Estrogen receptor polymorphism, estrogen content and idiopathic scoliosis in human: A possible genetic linkage. J. Steroid Biochem. Mol. Biol. 116, 56–60. 10.1016/j.jsbmb.2009.04.010 19406238

[B78] Faldini C. Manzetti M. Neri S. Barile F. Viroli G. Geraci G. (2022). Epigenetic and genetic factors related to curve progression in adolescent idiopathic scoliosis: A systematic scoping review of the current literature. Int. J. Mol. Sci. 23, 5914. 10.3390/ijms23115914 35682604 PMC9180299

[B79] Fiani B. Sekhon M. Doan T. Bowers B. Covarrubias C. Barthelmass M. (2021). Sacroiliac joint and pelvic dysfunction due to symphysiolysis in postpartum women. Cureus 13, e18619. 10.7759/cureus.18619 34786225 PMC8580107

[B80] Frank C. B. Shrive N. G. Boorman R. S. Lo I. K. Y. Hart D. a. (2004). New perspectives on bioengineering of joint tissues: Joint adaptation creates a moving target for engineering replacement tissues. Ann. Biomed. Eng. 32, 458–465. 10.1023/b:abme.0000017548.85451.b7 15095820

[B81] Fuentes N. Silveya P. (2019). Estrogen receptor signaling mechanisms. Adv. Protein Chem. Struct. Biol. 116, 135–170. 10.1016/bs.apcsb.2019.01.001 31036290 PMC6533072

[B82] Gardini E. S. Fiacco S. Mernone L. Ehlert U. (2020). Sleep and methylation of estrogen receptor genes, ESR1 and GPER, in healthy middle-aged and older women: Findings from the women 40+ healthy aging study. Nat. Sci. Sleep. 12, 525–536. 10.2147/NSS.S256102 32801978 PMC7394583

[B83] Gaulrapp H. Nuhrenborger C. (2022). The osgood-schlatter disease: A large clinical series with evaluation of risk factors, natural course, and outcomes. Int. Orthop. 46, 197–204. 10.1007/s00264-021-05178-z 34427770

[B84] Gegenhuber B. Tolkuhn J. (2022). Epigenetic mechanisms of brain sexual differentiation. Cold Spring Harb. Perspect. Biol. 14, a039099. 10.1101/cshperspect.a039099 35817509 PMC9620856

[B85] Ghasseminia S. Hareendranathan A. R. Jaremko J. L. (2021). Narrative review on the role of imaging in DDH. Indian J. Orthop. 55, 1456–1465. 10.1007/s43465-021-00511-5 35003536 PMC8688667

[B86] Ghasseminia S. Lim A. K. S. Concepcion N. D. P. Kirschner D. Teo Y. M. Dulai S. (2022). Interobserver variability of hip dysplasia indices on sweep ultrasound for novices, experts, and artificial intelligence. J. Pediatr. Orthop. 42, e315–e323. 10.1097/BPO.0000000000002065 35125417

[B87] Glass D. N. Giannini E. H. (1999). Juvenile rheumatoid arthritis as a complex genetic trait. Arthritis Rheum. 42, 2261–2268. 10.1002/1529-0131(199911)42:11<2261::AID-ANR1>3.0.CO;2-P 10555018

[B88] Godziuk K. Woodhouse L. J. Prado C. M. Forhan M. (2020). Clinical screening and identification of sarcopenic obesity in adults with advanced knee osteoarthritis. Clin. Nutr. ESPEN 40, 340–348. 10.1016/j.clnesp.2020.08.005 33183561

[B89] Gohar F. McArdle A. Jones M. Callan N. Hernandez B. Kessel C. (2019). Molecular signature characterisation of different inflammatory phenotypes of systemic juvenile idiopathic arthritis. Ann. Rheum. Dis. 78, 1107–1113. 10.1136/annrheumdis-2019.215051 31005900

[B90] Goldsmith L. T. Lust G. Steinetz B. G. (1994). Transmission of relaxin from lactating bitches to their offspring via suckling. Biol. Reprod. 50, 258–265. 10.1095/biolreprod50.2.258 8142544

[B91] Gorman K. F. Julien C. Moreau A. (2012). The genetic epidemiology of idiopathic scoliosis. Eur. Spine J. 21, 1905–1919. 10.1007/s00586-012-2389-6 22695700 PMC3463687

[B92] Gumucio J. P. Sugg K. B. Sibilsky Enselman E. R. Konja A. C. Eckhardt L. R. Bedi A. (2018). Anterior cruciate ligament tear induces a sustained loss of muscle fiber force production. Muscle Nerve 58, 145–148. 10.1002/mus.26075 PMC6051936 29346717

[B93] Haasnoot A. M. J. W. Kuiper J. J. W. de Boer J. H. (2019). Predicting uveitis in juvenile idiopathic arthritis: From biomarkers to clinical practice. Expert Rev. Clin. Immunol. 15, 657–666. 10.1080/1744666X.2019.1593139 30874452

[B94] Hale R. Hausselle J. G. Gonzalez R. V. (2014). A preliminary study on the differences in male and female muscle force distribution patterns during squatting and lunging maneuvers. Comput. Biol. Med. 52, 57–65. 10.1016/j.compbiomed.2014.06.010 25016289 PMC4156018

[B95] Hammes S. R. Levin E. R. (2019). Impact of estrogens in males and androgens in females. J. Clin. Invest. 129, 1818–1826. 10.1172/JCI125755 31042159 PMC6486327

[B96] Harmon K. G. Ireland M. L. (2000). Gender differences in noncontact anterior cruciate ligament injuries. Clin. Sports Med. 19, 287–302. 10.1016/s0278-5919(05)70204-0 10740760

[B97] Harsanyi S. Zamborsky R. Krajciova L. Kokavec M. Danisovic L. (2020). Developmental dysplasia of the hip: A review of etiopathogenesis, risk factors, and genetic aspects. Med. Kaunas. 56, 153. 10.3390/medicina56040153 PMC7230892 32244273

[B98] Hart D. A. (2018). Evidence for a potential “knee-eye-brain axis” involved in mobility and navigation control: Knee injury and obesity may disrupt axis integrity. J. Biomed. Sci. Eng. 11, 37–44. 10.4236/jbise.2018.113004

[B99] Hart D. A. Kydd A. Reno C. (1999). Gender and pregnancy affect neuropeptide responses of the rabbit Achilles tendon. Clin. Orthop. Relat. Res. 365, 237–246. 10.1097/00003086-199908000-00029 10627708

[B100] Hart D. A. (2021). Learning from human responses to deconditioning environments: Improved understanding of the “use it or lose it” principle. Front. Sports Act. Living 3, 685845. 10.3389/fspor.2021.685845 34927066 PMC8677937

[B101] Hart D. A. Natsu-ume T. Scoire P. Tasevski V. Frank C. B. Shrive N. G. (2002). Mechanobiology: Similarities and differences between *in vivo* and *in vitro* analysis at the functional and molecular levels. Recent Res. Devel Biophys. Biochem. 2, 153–177.

[B102] Hart D. A. (2022). Osteoarthritis as an umbrella term for different subsets of humans undergoing joint degeneration: The need to address the differences to develop effective conservative treatments and prevention strategies. Int. J. Mol. Sci. 23, 15365. In press. 10.3390/ijms232315365 36499704 PMC9736942

[B103] Hart D. A. Reno C. (1998). Pregnancy alters the *in vitro* responsiveness of the rabbit medial collateral ligament to neuropeptides: Effect on mRNA levels for growth factors, cytokines, iNOS, COX-2, metalloproteinases and TIMPs. Biochim. Biophys. Acta 1408, 35–43. 10.1016/s0925-4439(98)00051-9 9784599

[B104] Hart D. A. (2022). Sex differences in biological systems and the conundrum of menopause: Potential commonalities in post-menopausal disease mechanisms. Int. J. Mol. Sci. 23, 4119. 10.3390/ijms23084119 35456937 PMC9026302

[B105] Hart D. A. Werle J. Robert J. Kania-Richmond A. (2021). Long wait times for knee and hip total joint replacement in Canada: An isolated health problem, or a symptom of a larger problem? Osteoarthr. Cartil. Open 3, 100141. 10.1016/j.ocarto.2021.100141 36474990 PMC9718171

[B106] Herzberg S. D. Motuapuaka M. L. Lambert W. Fu R. Brady J. Guise J. M. (2017). The effect of menstrual cycle and contraceptives on ACL injuries and laxity: A systematic review and meta-analysis. Orthop. J. Sports Med. 5, 2325967117718781. 10.1177/2325967117718781 28795075 PMC5524267

[B107] Hewett T. E. Myer G. D. Kiefer A. W. Ford K. R. (2015). Longitudinal increases in knee abduction moments in females during adolescent growth. Med. Sci. Sports Exerc. 47, 2579–2585. 10.1249/MSS.0000000000000700 25970663 PMC4643418

[B108] Hewett T. E. Roewer B. Ford K. Myer G. (2015). Multicenter trial of motion analysis for injury risk prediction: Lessons learned from prospective longitudinal large cohort combined biomechanical -epidemiological studies. Braz. J. Phys. Ther. 19, 398–409. 10.1590/bjpt-rbf.2014.0121 26537810 PMC4647151

[B109] Hinks A. Marion M. Cobb J. Comeau M. E. Sudman M. Ainsworth H. C. (2018). Brief report: The genetic profile of rheumatoid factor-positive polyarticular juvenile idiopathic arthritis resembles that of adult rheumatoid arthritis. Arthritis Rheumatol. 70, 957–962. 10.1002/art.40443 29426059 PMC5984672

[B110] Hollenbach J. A. Thompson S. D. Bugawan T. L. Ryan M. Sudman M. Marion M. (2010). Juvenile idiopathic arthritis and HLA class I and class II interactions and age-at-onset effects. Arthritis Rheum. 62, 1781–1791. 10.1002/art.27424 20191588 PMC2917207

[B111] Ipulan-Colet L. A. (2022). Sexual dimorphism through androgen signaling, from external genitalia to muscles. Front. Endocrinol. (Lausanne) 13, 940229. 10.3389/fendo.2022.940229 35983512 PMC9379613

[B112] Ireland M. L. (2002). The female ACL: Why is it more prone to injury? Orthop. Clin. North Am. 33, 637–651. 10.1016/s0030-5898(02)00028-7 12528906

[B113] Jacobsen S. Sonne-Holm S. Rovsing H. Monrad H. Gebuhr P. (2007). Degenerative lumbar spondylolisthesis: An epidemiological perspective: The copenhagen osteoarthritis study. Spine (Phila Pa 1776) 32, 120–125. 10.1097/01.brs.0000250979.12398.96 17202902

[B114] Jia S. Zhao W. Hu F. Zhao Y. Ge M. Xia X. (2022). Sex differences in the association of physical activity levels and vitamin D with obesity, sarcopenia, and sarcopenic obesity: A cross-sectional study. BMC Geriatr. 22, 898. 10.1186/s12877-022-03577-4 36434519 PMC9701059

[B115] Jin L. Y. Song X. X. Li X. F. (2020). The role of estrogen in intervertebral disc degeneration. Steroids 154, 108549. 10.1016/j.steroids.2019.108549 31812622

[B116] Johnson A. K. Palmieri R. M. Lepley L. K. (2018). Contribution of neuromuscular factors to quadriceps asymmetry after anterior cruciate ligament reconstruction. J. Athl. Train. 53, 347–354. 10.4085/1062-6050-463-16 29652169 PMC5967276

[B117] Johnston C. B. Dagar M. (2020). Osteoporosis in older adults. Med. Clin. North Am. 104, 873–884. 10.1016/j.mcna.2020.06.004 32773051

[B118] Joyner M. J. Barnes J. N. Hart E. C. Wallin B. G. Charkoudian N. (2015). Neural control of the circulation: How sex and age differences interact in humans. Compr. Physiol. 5, 198–215. 10.1002/cphy.c.140005 PMC4459710 25589269

[B119] Kaelin A. J. (2020). Adolescent idiopathic scoliosis: Indications for bracing and conservative treatments. Ann. Transl. Med. 8, 28. 10.21037/atm.2019.09.69 32055619 PMC6995912

[B120] Kaji H. (2013). Linkage between muscle and bone: Common catabolic signals resulting in osteoporosis and sarcopenia. Curr. Opin. Clin. Nutr. Metab. Care. 16, 272–277. 10.1097/MCO.0b013e32835fe6a5 23481148

[B121] Kaplan Y. (2011). Identifying individuals with an anterior cruciate ligament-deficient knee as copers and noncopers: A narrative literature review. J. Orthop. Sports Phys. Ther. 41, 758–766. 10.2519/jospt.2011.3384 21979555

[B122] Kilsdonk I. Witbreuk M. Van Den Woude H. J. (2021). Ultrasound of the neonatal hip as a screening tool for DDH: How to screen and differences in screening programs between European countries. J. Ultrason. 21, e147–e153. 10.15557/JoU.2021.0024 34258040 PMC8264809

[B123] Klassen S. A. Joyner M. J. Baker S. E. (2021). The impact of aging and sex on sympathetic neurocirculatory regulation. Semin. Cell Dev. Biol. 116, 72–81. 10.1016/j.semcdb.2021.01.001 33468420 PMC8282778

[B124] Komut E. Zehir S. (2021). Can developmental dysplasia of the hip be identified in the prenatal period? A pilot study: Ultrasonographic evaluation and postnatal follow-up results of fetal hips in the third trimester. Acta Orthop. Traumatol. Turc 55, 196–200. 10.5152/j.aott.2021.20143 34100358 PMC10566353

[B125] Koolschijn P. C. M. P. Crone E. A. (2013). Sex differences and structural brain maturation from childhood to early adulthood. Dev. Cogn. Neurosci. 5, 106–118. 10.1016/j.dcn.2013.02.003 23500670 PMC6987760

[B126] Kosar S. N. Guzel Y. Kose M. G. Islker A. K. Hazr T. (2022). Whole and segmental body composition changes during mid-follicular and mid-luteal phases of the menstrual cycle in recreationally active young women. Ann. Hum. Biol. 49, 124–132. 10.1080/03014460.2022.2088857 35696275

[B127] Kovacs T. Szabo-Meleg E. Abraham I. M. (2020). Estradiol-induced epigenetically mediated mechanisms and regulation of gene expression. Int. J. Mol. Sci. 21, 3177. 10.3390/ijms21093177 32365920 PMC7246826

[B128] Kruger T. H. C. Leeners B. Tronci E. Mancini T. Ille F. Egli M. (2023). The androgen system across the menstrual cycle: Hormonal, (epi)genetic and psychometric alterations. Physiol. Behav. 259, 114034. 10.1016/j.physbeh.2022.114034 36403781

[B129] Kudo D. Miyakoshi N. Hongo M. Matsumoto-Miyai K. Kasukawa Y. Misawa A. (2015). Nerve growth factor and estrogen receptor mRNA expression in paravertebral muscles of patients with adolescent idiopathic scoliosis: A preliminary study. Spine Deform. 3, 122–127. 10.1016/j.jspd.2014.07.006 27927302

[B130] Kulis A. Gozdzialska A. Drag J. Jaskiewicz J. Knapik-Czajka M. Lipik E. (2015). Participation of sex hormones in multifactorial pathogenesis of adolescent idiopathic scoliosis. Int. Orthop. 39, 1227–1236. 10.1007/s00264-015-2742-6 25804208

[B131] Kuntze G. Nesbitt C. Nettel-Aguirre A. Esau S. Scholz R. Brooks J. (2020). Gait adaptations in youth with juvenile idiopathic arthritis. Arthritis Care Res. Hob. 72, 917–924. 10.1002/acr.23919 31058454

[B132] Kuznia A. L. Hernandez A. K. Lee L. U. (2020). Adolescent idiopathic scoliosis: Common questions and answers. Am. Fam. Physician 101, 19–23. 31894928

[B133] Kydd A. S. Reno C. R. Tsao H. W. Hart D. A. (2007). Impact of age, systemic glucocorticoids, and progressive knee arthritis on specific mRNA levels in different areas of the rabbit cornea. Cornea 26, 352–361. 10.1097/ICO0b013e318033a534 17413965

[B134] Lawra J. Conrad S. Chafetz R. S. Boniello M. Franklin C. (2021). Stiff landings, core stability, and dynamic knee valgus: A systematic review on documented anterior cruciate ligament ruptures in male and female athletes. Int. J. Environ. Res. Public Health 18, 3826. 10.3390/ijerph18073826 33917488 PMC8038785

[B135] Lee C. A. Lee-Barthel A. Marquino L. Sandoval N. Marcotte G. R. Baar K. (2015). Estrogen inhibits lysyl oxidase and decreases mechanical function in engineered ligaments. J. Appl. Physiol. 118, 1250–1257. 10.1152/japplphysiol.00823.2014 25979936

[B136] Lee C. B. Mata-Fink A. Mills M. B. Kim Y. J. (2013). Demographic differences in adolescent-diagnosed and adult-diagnosed acetabular dysplasia compared with infantile developmental dysplasia of the hip. J. Pediatr. Orthop. 33, 107–111. 10.1097/BPO.0b013e3182745456 23389561

[B137] Lee H. I. Kim H. J. Jo S. Shim S. C. Kim T. H. Won E. J. (2022). IL-6 activates pathologic Th17 cell via STAT3 phosphorylation in inflammatory joint of ankylosing spondylitis. Biochem. Biophys. Res. Commun. 620, 69–75. 10.1016/j.bbrc.2022.06.081 35780583

[B138] Lee H. Petrofsky J. S. Daher N. Berk L. Laymon M. Khowailed I. A. (2013). Anterior cruciate ligament elasticity and force for flexion during the menstrual cycle. Med. Sci. Monit. 19, 1080–1088. 10.12659/MSM.889393 24287619 PMC3862144

[B139] Lee J. J. Y. Duffy C. M. Guzman J. Oen K. Barrowman N. Rosenberg A. M. (2019). Prospective determination of the incidence and risk factors of new-onset uveitis in juvenile idiopathic arthritis: The research in arthritis in Canadian children emphasizing outcomes cohort. Arthritis Care Res. Hob. 71, 1436–1443. 10.1002/acr.23783 30320957

[B140] Lee W. C. Lee P. A. Chen T. Y. Wang T. M. Lu T. W. (2022). Bilateral asymmetry in balance control during gait in children with treated unilateral developmental dysplasia of the hip. Gait Posture 92, 223–229. 10.1016/j.gaitpost.2021.11013 34871927

[B141] Li X. Liang A. Cui Y. Liao J. Fang X. Zhong S. (2022). Role of macrophage-associated chemokines in the assessment of initial axial spondyloarthritis. Clin. Rheumatol. 41, 3383–3389. 10.1007/s10067-022-06308-7 35882716

[B142] Liang Z. T. Guo C. T. Li J. Zhang H. Q. (2021). The role of endocrine hormones in the pathogenesis of adolescent idiopathic scoliosis. FASEB J. 35, e21839. 10.1096/fj.202100759R 34387890

[B143] Lim S. Y. (2022). Romosozumab for the treatment of osteoporosis in women: Efficacy, safety, and cardiovascular risk. Women’s health (Lond) 18, 17455057221125577. 10.1177/17455057221125577 36154750 PMC9511529

[B144] Lin C. Y. Casey E. Herman D. C. Katz N. Tenforde A. S. (2018). Sex differences in common sports injuries. PM R. 10, 1073–1082. 10.1016/j.pmrj.2018.03.008 29550413 PMC6138566

[B145] Liu S. H. al-Shaikh R. Panossian V. Yang R. S. Nelson S. D. Soleiman N. (1996). Primary immunolocalization of estrogen and progesterone target cells in the human anterior cruciate ligament. J. Orthop. Res. 14, 526–533. 10.1002/jor.1100140405 8764860

[B146] Lohmander L. S. Englund P. M. Dahl L. L. Roos E. M. (2007). The long-term consequence of anterior cruciate ligament and meniscus injuries: Osteoarthritis. Am. J. Sports Med. 35, 1756–1769. 10.1177/0363546507307396 17761605

[B147] Lu T. Achari Y. Sciore P. Hart D. A. (2006). Estrogen receptor alpha regulates matrix metalloproteinase-13 promoter activity primarily through the AP-1 transcriptional regulatory site. Biochim. Biophys. Acta 1762, 719–731. 10.1016/j.bbadis.2006.06.007 16919424

[B148] Lumsden M. A. Sassarini J. (2019). The evolution of the human menopause. Climacteric 22, 111–116. 10.1080/13697137.2018.1547701 30712396

[B149] Madeira M. Mattar A. Logullo A. F. Soares F. A. Gebrim L. H. (2013). Estrogen receptor alpha/beta ratio and estrogen receptor beta as predictors of endocrine therapy responsiveness-a randomized neoadjuvant trial comparison between anastrozole and tamoxifen for the treatment of postmenopausal breast cancer. BMC Cancer 13, 425. 10.1186/1471-2407-13-425 24047421 PMC3851532

[B150] Mahboobfard F. Pourgholami M. H. Jorjani M. Dargahi L. Amiri M. Sadeghi S. (2022). Estrogen as a key regulator of energy homeostasis and metabolic health. Biomed. Pharmacother. 156, 113808. 10.1016/j.biopha.2022.113808 36252357

[B151] Maniar N. Cole M. H. Bryant A. L. Opar D. A. (2022). Muscle force contributions to anterior cruciate ligament loading. Sports Med. 52, 1737–1750. 10.1007/s40279-022-01674-3 35437711 PMC9325827

[B152] Marotta N. Demeco A. de Scorpio G. Indino A. Iona T. Ammendola A. (2019). Late activation of the vastus medialis in determining the risk of anterior cruciate ligament injury in soccer players. J. Sport Rehabil. 29, 952–955. 10.1123/jsr.2019-0026 31711040

[B153] Marty-Poumarat C. Ostertag A. Baudoin C. Marpeau M. de Vernejoul M. C. Cohen-Solal M. (2012). Does hormone replacement therapy prevent lateral rotatory spondylolisthesis in postmenopausal women? Eur. Spine J. 21, 1127–1134. 10.1007/s00586-011-2048-3 22033571 PMC3366144

[B154] Maruyama S. Sekine C. Shagawa M. Yokota H. Hirabayashi R. Togashi R. (2022). Menstrual cycle changes joint laxity in females-differences between eumenorrhea and oligomenorrhea. J. Clin. Med. 11, 3222. 10.3390/jcm11113222 35683609 PMC9181714

[B155] Maruyama S. Yamazaki T. Sato Y. Suzuki Y. Shimizu S. Ikezu M. (2021). Relationship between anterior knee laxity and general joint laxity during the menstrual cycle. Orthop. J. Sports Med. 9, 2325967121993045. 10.1177/2325967121993045 33855094 PMC8010836

[B156] Marzo-Ortega H. Navarro-Compan V. Akar S. Kiltz U. Clark Z. Nikiphorou E. (2022). The impact of gender and sex on diagnosis, treatment outcomes and health-related quality of life in patients with axial spondyloarthritis. Clin. Rheumatol. 41, 3573–3581. 10.1007/s10067-022-06228-6 35763155 PMC9568456

[B157] Masi A. T. (1992). Do sex hormones play a role in ankylosing spondylitis? Rheum. Dis. Clin. North Am. 18, 153–176. 10.1016/s0889-857x(21)00715-8 1561401

[B158] Mattu A. T. Ghali B. Linton V. Zheng A. Pike I. (2022). Prevention of non-contact anterior cruciate ligament injuries among youth female athletes: An umbrella review. Int. J. Environ. Res. Public Health 19, 4648. 10.3390/ijerph19084648 35457516 PMC9027388

[B159] Mayayo-Peralta I. Prekovic S. Zwart W. (2021). Estrogen receptor on the move: Cistromic plasticity and its implications in breast cancer. Mol. Asp. Med. 78, 100939. 10.1016/j.mam.2020.100939 33358533

[B160] McDougall J. J. Bray R. C. Hart D. A. (2000). Late gestational changes in sympathomimetic sensitivity in primagravid rabbit ligaments. Can. J. Physiol. Pharmacol. 78, 528–534. 10.1139/y00-020 10926158

[B161] McDougall J. J. Giles R. W. Bray R. C. Hart D. A. (1998). Pregnancy-induced changes in rabbit medial collateral ligament vasoregulation. Am. J. Physiol. 275, R1380–R1385. 10.1152/ajpregu.1998.275.4.R1380 9756572

[B162] McMillian S. L. Minchew E. C. Lowe D. A. Spangerburg E. E. (2022). Skeletal muscle wasting: The estrogen side of sexual dimorphism. Am. Rev. Physiol. Cell Physiol. 322, C24–C37. 10.1152/ajpcell.00333.2021 PMC8721895 34788147

[B163] Mease P. J. McLean R. R. Dube B. Liu M. Rebello S. Glynn M. (2021). Comparison of men and women with axial spondyloarthritis in the US-based corrona psoriatic arthritis/spondyloarthritis registry. J. Rheumatol. 48, 1528–1536. 10.3899/jrheum.201549 33858974

[B164] Monasterio X. Gil S. M. Bidaurrazaga-Letona I. Lekue J. A. Santisteban J. M. Diaz-Beitia G. (2021). The burden of injuries according to maturity status and timing: A two-decade study with 110 growth curves in an elite football academy. Eur. J. Sport Sci. 13, 267–277. 10.1080/17461391.2021.2006316 34767492

[B165] Monotas M. C. Gonzalez D. M. Cespedes C. Forero C. Mareno A. P. R. (2022). Genetic and epigenetic control of puberty. Sex. Dev. 16, 1–10. 10.1159/000519039 34649256 PMC8820423

[B166] Mousavibaygei S. R. Karimnia a. Germami M. H. Azadmehr F. Erfanifam T. Ghaedi A. (2022). An evaluation of clinical and ultrasound results of Pavlik harness treatment for developmental dysplasia of the hip. J. Med. Life 15, 850–853. 10.25122/jml-2021-0289 35928353 PMC9321489

[B167] Muellner M. Kreutzinger V. Becker L. Diekhoff T. Bumberger M. Schomig F. (2022). Unexpected sex differences in the relationship of sacroiliac joint and lumbar spine degeneration. Diagn. (Basal) 12, 275. 10.3390/diagnostics12020275 PMC8870885 35204366

[B168] Mulder J. E. Kolatkar N. S. LeBoff M. S. (2016). Drug insight: Existing and emerging therapies for osteoporosis. Nat. Clin. Pract. Endocrinol. Metab. 2, 670–680. 10.1038/ncpendmet0325 17143314

[B169] Muollo V. Tatangelo T. Ghiotto L. Cavedon V. ilanese C. Zamboni M. (2022). Is handgrip strength a marker of muscle and physical function of the lower limbs? Sex differences in older adults with obesity. Nutr. Metab. Cardiovasc Dis. 32, 2168–2176. 10.1016/j.numecd.2022.06.018< 35850750

[B170] Murphy P. G. Hart D. A. (1993). Plasminogen activators and plasminogen activator inhibitors in connective tissues and connective tissue cells: Influence of the neuropeptide substance P on expression. Biochim. Biophys. Acta 1182, 205–214. 10.1016/0925-4439(93)90142-n 7689342

[B171] Musahl V. Karlsson J. (2019). Anterior cruciate ligament tear. N. Engl. J. Med. 380, 2341–2348. 10.1056/NEJMcp1805931 31189037

[B172] Myklebust G. Engebretsen L. Braekken I. H. Skjolberg A. Olsen O. E. Bahr R. (2007). Prevention of noncontact anterior cruciate ligament injuries in elite and adolescent female team handball athletes. Instr. Course Lect. 56, 407–418. 17472324

[B173] Myklebust M. Skjolberg A. Bahr R. (2013). ACL injury incidence in female handball 10 years after the Norwegian ACL prevention study: Important lessons learned. Br. J. Sports Med. 47, 476–479. 10.1136/bjsports-2012-091862 23403528

[B174] Nam J. H. Koh Y. G. Kim P. S. Kang K. Park J. H. Kang K. T. (2020). Gender difference in bowing of the sagittal femoral morphology measurement using magnetic resonance imaging. Surg. Radiol. Anat. 42, 1231–1236. 10.1007/s00276-020-02488-6 32405786

[B175] Neuhaus C. Appenzeller-Herzog C. Faude O. (2021). A systematic review on conservative treatment options for Osgood-Schlatter disease. Phys. Ther. Sport 49, 178–187. 10.1016/j.ptsp.2021.03.002 33744766

[B176] Nieves J. W. (2017). Sex-differences in skeletal growth and aging. Curr. Osteoporos. Rep. 15, 70–75. 10.1007/s11914-017-0349-0 28181064

[B177] Nigrovic P. A. Colbert R. A. Holers V. M. Ozen S. Ruperto N. Thompson S. D. (2021). Biological classification of childhood arthritis: Roadmap to a molecular nomenclature. Nat. Rev. Rheumatol. 17, 257–269. 10.1038/s41584-021-00590-6 33731872 PMC10355214

[B178] Onel K. B. Horton D. B. Lovell D. J. Sjenoi S. Cuello C. A. Amgles-Han S. T. (2021). 2021 American college of rheumatology guideline for the treatment of juvenile idiopathic arthritis: Therapeutic approaches for oligoarthritis, temporomandibular joint arthritis, and systemic juvenile idiopathic arthritis. Arthritis Rheumatol. 74, 553–569. 10.1002/art.42037 PMC10161784 35233993

[B179] Oosthuyse T. Bosch A. N. (2010). The effect of the menstrual cycle on exercise metabolism: Implications for exercise performance in eumenorrhoeic women. Sports Med. 40, 207–227. 10.2165/11317090-000000000-00000 20199120

[B180] Oveisgharan S. Yang J. Yu L. Burba D. Bang W. Tasaki S. (2023). Estrogen receptor genes, cognitive decline, and Alzheimer disease. Neurology 100, e1474–e1487. 10.1212/wnl.0000000000206833 36697247 PMC10104608

[B181] Pan M. Pan X. Zhou J. Wang J. Qi Q. Wang L. (2022). Update on hormone therapy for the management of postmenopausal women. Biosci. Trends 16, 46–57. 10.5582/bst.2021.01418 35013031

[B182] Park S. C. Son S. W. Yang J. H. Chang D. G. Suh S. W. Nam Y. (2022). Novel surgical technique for adolescent idiopathic scoliosis: Minimally invasive scoliosis surgery. J. Clin. Med. 11, 5847. 10.3390/jcm11195847 36233714 PMC9572236

[B183] Park S. K. Stefanyshyn D. J. Loitz-Ramage B. Hart D. A. Ronsky J. L. (2009). Changing hormone levels during the menstrual cycle affect knee laxity and stiffness in healthy female subjects. Am. J. Sports. Med. 37, 588–598. 10.1177/0363546508326713 19174550

[B184] Park Y. Ko J. Y. Jang J. Y. Lee S. Beom J. Ryu J. S. (2021). Asymmetrical activation and asymmetrical weakness as two different mechanisms of adolescent idiopathic scoliosis. Sci. Rep. 11, 17582. 10.1038/s41598-021-96882-8 34475442 PMC8413345

[B185] Parker E. A. Meyer A. M. Fleury I. G. Buckwalter J. A. 5th (2022). Menstrual hormone-induced cyclic thumb CMC instability and degeneration in women: A systematic review. Biol. Sex. Diff 13, 32. 10.1186/s13293-022-00438-y PMC9208132 35725646

[B186] Penha P. J. Penha Ramos N. L. J. de Carvalho B. K. G. Andrade R. M. Schmitt A. C. B. Joao S. M. A. (2018). Prevalence of adolescent idiopathic scoliosis in the state of Sao Paulo, Brazil. Spine (Phila Pa 1976) 43, 1710–1718. 10.1097/BRS.0000000000002725 29877996

[B187] Penolazzi L. Lambertini E. Giordano S. Sollazzo V. Traina G. del Senno L. (2004). Methylation analysis of the promoter F of estrogen receptor alpha gene: Effects on the level of transcription on human osteoblastic cells. J. Steroid Biochem. Mol. Biol. 91, 1–9. 10.1016/j.jsbmb.2004.02.005 15261302

[B188] Perruccio A. V. Badley E. M. Power J. D. Canizares M. Kapoor M. Rockel J. (2019). Sex differences in the relationship between individual systemic markers of inflammation and pain in knee osteoarthritis. Osteoarthr. Cartil. Open 1, 100004. 10.1016/j.ocarto.2019.100004 36474721 PMC9718173

[B189] Petersen D. N. Tkalcevic G. T. Koza-Taylor P. H. Turi T. G. Brown T. A. (1998). Identification of estrogen receptor beta2, a functional variant of estrogen receptor beta expressed in normal rat tissues. Endocrinology 139, 1082–1092. 10.1210/endo.139.3.5840 9492041

[B190] Pezaro S. Pearce G. Reinhold E. (2020). Understanding hypermobile ehlers-danlos syndrome and hypermobility spectrum disorders in the context of childbearing: An international qualitative study. Midwifery 88, 102749. 10.1016/j.midw.2020.102749 32535291

[B191] Pollak L. Shlamkovic N. Minewicz A. Mirovsky Y. (2013). Otolith dysfunction as a possible cause for the development of idiopathic scoliosis. J. Pediatr. Orthop. 33, 293–297. 10.1097/BPO.0b013e31827c0643 23482266

[B192] Pujol P. Rey M. Nirde P. Roger P. Gastaldi M. Laffargue F. (1998). Differential expression of estrogen receptor-alpha and -beta messenger RNAs as a potential marker of ovarian carcinogenesis. Cancer Res. 58, 5367–5373. 9850067

[B193] Quartier P. (2021). Juvenile idiopathic arthritis-associated chronic uveitis: Recent therapeutic approaches. J. Clin. Med. 10, 2934. 10.3390/jcm10132934 34208973 PMC8269439

[B194] Quatman C. E. Ford K. R. Myer G. D. Hewett T. E. (2006). Maturation leads to gender differences in landing force and vertical jump performance: A longitudinal study. Am. J. Sports Med. 34, 806–813. 10.1177/0363546505281916 16382009

[B195] Raczkowski J. W. (2007). The concentrations of testosterone and estradiol in girls with adolescent idiopathic scoliosis. Neuro Endocrinol. Lett. 28, 302–304. 17627266

[B196] Radin E. L. Burr D. B. Caterson B. Fyhrie D. Brown T. D. Boyd R. D. (1991). Mechanical determinants of osteoarthrosis. Semin. Arthritis Rheum. 21, 12–21. 10.1016/0049-0172(91)90036-y 1796301

[B197] Raggio C. L. (2006). Sexual dimorphism in adolescent idiopathic scoliosis. Orthop. Clin. N. Am. 37, 555–558. 10.1016/j.ocl.2006.09.010 17141012

[B198] Ramsay D. J. (1989). The importance of thirst in maintenance of fluid balance. Baillieres Clin. Endocrinol. Metab. 3, 371–391. 10.1016/s0950-351x(89)80008-4 2698142

[B199] Renstrom P. Ljunggvist A. Arendt E. Beynnon B. Fukubayashi T. Garrett W. (2008). Non-contact ACL injuries in female athletes: An international olympic committee current concepts statement. Br. J. Sports Med. 42, 394–412. 10.1136/bjsm.2008.048934 18539658 PMC3920910

[B200] Ringold S. Beukelman T. Nigrovic P. A. Kimura Y. Carra Registry Site (2013). Race, ethnicity, and disease outcomes in juvenile idiopathic arthritis: A cross-sectional analysis of the childhood arthritis and rheumatology research alliance (CARRA) registry. J. Rheumatol. 40, 936–942. 10.3899/jrheum.121147 23588937

[B201] Rinonapoli G. Ruggiero C. Meccariello L. Bisaccia M. Ceccarini P. Caraffa A. (2021). Osteoporosis in men: A review of an underestimated bone condition. Int. J. Mol. Sci. 22, 2105. 10.3390/ijms22042105 33672656 PMC7924179

[B202] Robinson H. S. Lindgren A. Bjelland E. K. (2022). Generalized joint hypermobility and risk of pelvic girdle pain in pregnancy: Does body mass index matter? Physiother. Theory Pract. 38, 2222–2229. 10.1080/09593985.2021.1913774 33849378

[B203] Rosenberg A. M. (2002). Uveitis associated with childhood rheumatic diseases. Curr. Opin. Rheumatol. 14, 542–547. 10.1097/00002281-200209000-00011 12192252

[B204] Rosenberg A. M. (1987). Uveitis associated with juvenile rheumatoid arthritis. Semin. Arthritis Rheum. 16, 158–173. 10.1016/0049-0172(87)90019-9 3547655

[B205] Rumancik S. Routh R. H. Tathak R,D. Burshell A. L. Nauman E. A. (2005). Assessment of bone quantity and distribution in adult lumbar scoliosis: New dual-energy x-ray absorptiometry methodology and analysis. Spine (Phila PA 1776) 30, 434–439. 10.1097/01.brs.0000153344.06682.b2 15706341

[B206] Rusin B. Kotwicki T. Glodek A. Andrusiewicz M. Urbariak P. Kotwicka M. (2012). Estrogen receptor 2 expression in back muscles of girls with idiopathic scoliosis-relation to radiological parameters. Stu Healthy Technol. Inf. 176, 59–62. 22744458

[B207] Sadoghi P. von Keudell A. Vavken P. (2012). Effectiveness of anterior cruciate ligament injury prevention training programs. J. Bone Jt. Surg. Am. 94, 769–776. 10.2106/JBJS.K.00467 22456856

[B208] Saks B. R. Fox J. D. Owens J. S. Maldonado D. R. Jimenez A. E. Ankem H. K. (2021). One bony morphology, two pathologic entities: Sex-based differences in patients with borderline hip dysplasia undergoing hip arthroscopy. Am. J. Sports Med. 49, 3906–3914. 10.1177/03635465211043510 34694159

[B209] Salo P. Bray R. Seerattan R. Reno C. McDougall J. Hart D. A. (2007). Neuropeptides regulate expression of matrix molecule, growth factor and inflammatory mediator mRNA in explants of normal and healing medial collateral ligament. Regul. Pept. 142, 1–6. 10.1016/j.regpep.2007.01.001 17292490

[B210] Santos F. F. Lourenco B. M. Souza M. B. Maia L. B. Olivereira V. C. Olivereira M. X. (2022). Prevention of low back and pelvic girdle pain during pregnancy: A systematic review and meta-analysis of randomised controlled trials with GRADE recommendations. Physiotherapy 118, 1–11. 10.1016/j.physio.2022.09.004 36288631

[B211] Schauberger C. W. Rooney B. L. Goldsmith L. Shenton D. Silva P. D. Schaper A. (1996). Peripheral joint laxity increases in pregnancy but does not correlate with serum relaxin levels. Am. J. Obstet. Gynecol. 174, 667–671. 10.1016/s0002-9378(96)70447-7 8623804

[B212] Schmitz R. J. Shultz S. J. (2013). Anterior knee stiffness changes in laxity “responders” versus “nonresponders” across the menstrual cycle. J. Athl. Train. 48, 39–46. 10.4085/1062-6050-47.6.07 23672324 PMC3554031

[B213] Sciore P. Frank C. B. Hart D. A. (1998). Identification of sex hormone receptors in human and rabbit ligaments of the knee by reverse transcription-polymerase chain reaction: Evidence that receptors are present in tissue from both male and female subjects. J. Orthop. Res. 16, 604–610. 10.1002/jor.1100160513 9820285

[B214] Scott J. R. Muangman P. Gibran N. S. (2007). Making sense of hypertrophic scar: A role for nerves. Wound Repair Regen. 15, 527–S31. 10.1111/j.1524-475X.2007.00222.x 17727464

[B215] Seleviciene V. Cesnaviciute A. Strukcinskiene B. marcinowicz L. Strazdiene N. Genowska A. (2022). Physiotherapeutic scoliosis-specific exercise methodologies used for conservative treatment of adolescent idiopathic scoliosis, and their effectiveness: An extended literature review of current research and practice. Int. J. Environ. Res. Public Health 19, 9240. 10.3390/ijerph19159240 35954620 PMC9368145

[B216] Serhal L. Lwin M. N. Holroyd C. Edwards C. J. (2020). Rheumatoid arthritis in the elderly: Characteristics and treatment considerations. Autoimmun. Rev. 19, 102528. 10.1016/j.autrev.2020.102528 32234572

[B217] Seve P. Kodjikian L. Adelaide L. Jamilloux Y. (2015). Uveitis in adults: What do rheumatologists need to know? Jt. Bone Spine 82, 308–314. 10.1016/j.jbspin.2015.06.002 26184541

[B218] Shagawa M. Maruyama S. Sekine C. Yokota H. Hirabayashi R. Hirata A. (2021). Comparison of anterior knee laxity, stiffness, genu recurvatum, and general joint laxity in the late follicular phase and the ovulatory phase of the menstrual cycle. BMC Musculoskelet. Disord. 22, 886. 10.1186/s12891-021-04767-8 34663291 PMC8524894

[B219] Shah L. Elshaikh A. O. Lee R. Mathew C. J. Jose M. T. Cancarevic I. (2020). Cancarevic. Do menopause and aging affect the onset and progression of rheumatoid arthritis and systemic lupus erythematosus? Cureus 12, e10944. 10.7759/cureus.10944 33072443 PMC7557711

[B220] Sharip A. Kunz J. (2020). Understanding the pathogenesis of spondyloarthritis. Biomolecules 10, 1461. 10.3390/biom10101461 33092023 PMC7588965

[B221] Shaw B. A. Segal L. S. Section on Orthopaedics (2016). Evaluation and referral for developmental dysplasia of the hip in infants. Pediatrics 138, e20163107. 10.1542/peds.2016-3107 27940740

[B222] Sheng F. Xia C. Xu L. Qin X. Tang N. L. S. Qiu Y. (2019). New evidence supporting the role of FBN1 in the development of adolescent idiopathic scoliosis. Spine (Phila PA 1776) 44, E225–E232. 10.1097/BRS.0000000000002809 30044367

[B223] Shepherd R. Cheung A. S. Pang K. Saffery R. Novakovic B. (2021). Sexual dimorphism in innate immunity: The role of sex hormones and epigenetics. Front. Immunol. 11, 604000. 10.3389/fimmu.2020.604000 33584674 PMC7873844

[B224] Shultz S. J. Wideman L. Montgomery M. M. Beasley K. N. Nindl B. C. (2012). Changes in serum collagen markers, IGF-I, and knee joint laxity across the menstrual cycle. J. Orthop. Res. 30, 1405–1412. 10.1002/jor.22093 22389002 PMC3371148

[B225] Skogland L. B. Miller J. A. (1980). Growth related hormones in idiopathic scoliosis. An endocrine basis for accelerated growth. Acta Orthop. Scand. 51, 779–780. 10.3109/17453678008990874 6781216

[B226] Somerson J. S. Isby I. J. Hagen M. S. Kweson C. Y. Gee A. O. (2019). The menstrual cycle may affect anterior knee laxity and the rate of anterior cruciate ligament rupture: A systematic review and meta-analysis. JBJS Rev. 7, e2. 10.2106/JBJS.RVW.18.00198 31490339

[B227] Song R. X-D. Santen R. J. (2006). Membrane initiated estrogen signaling in breast cancer. Biol. Reprod. 75, 9–16. 10.1095/biolreprod.105.050070 16571873

[B228] Springer B. Bechler U. Waldstein W. Rueckl K. Boettner C. S. Boettner F. (2020). The influence of femoral and tibial bony anatomy on valgus OA of the knee. Knee Surg. Sports Traumatol. Arthrosc. 28, 2998–3006. 10.1007/s00167-019-05734-6 31595340

[B229] Steinmetz B. G. Goldsmith L. T. Lust G. (1987). Plasma relaxin levels in pregnant and lactating dogs. Biol. Reprod. 37, 719–725. 10.1095/biolreprod37.3.719 3676415

[B230] Stepien A. Maslanko K. Rekowski W. Fabian K. Tuz J. Graff K. (2022). Analysis of the prevalence of asymmetry and muscle tone disorders in the first year of life among youth with idiopathic scoliosis: A retrospective case-control study. J. Back Musculoskelet. Rehabil. 35, 1003–1011. 10.3233/BMR-171075 35431225

[B231] Stovall R. ven den Horst-Bruinsma I. E. Liu S. H. Rusman T. Gensler L. S. (2022). Sexual dimorphism in the prevalence, manifestation and outcomes of axial spondyloarthritis. Nat. Rev. Rheumatol. 18, 657–669. Dpoi:. 10.1038/s41584-022-00833-0 36109666

[B232] Sugihara T. (2022). Treatment strategies for elderly-onset rheumatoid arthritis in the new era. Mod. Rheumatol. 32, 493–499. 10.1093/mr/roab087 34791359

[B233] Sutton K. M. Bullock J. M. (2013). Anterior cruciate ligament rupture: Differences between males and females. J. Am. Acad. Orthop. Surg. 21, 41–50. 10.5435/JAAOS-21-01-41 23281470

[B234] Swarup I. Penny C. L. Dodwell E. R. (2018). Developmental dysplasia of the hip: An update on diagnosis and management from birth to 6 months. Curr. Opin. Pediactr 30, 84–92. 10.1097/MOP.0000000000000574 29194074

[B235] Szyf M. (2021). Perinatal stress and epigenetics. Handb. Clin. Neurol. 180, 125–148. 10.1016/B978-0-12-820107-7-00008-2 34225925

[B236] Taradaj K. Ginda T. Maciejewicz P. Ciechanowicz P. Suchonska B. Hajbos M. (2018). Pregnancy and the eye. Changes in morphology of the cornea and the anterior chamber of the eye in pregnant woman. Ginekol. Pol. 89, 695–699. 10.5603/GP.a2018.0117 30618038

[B237] Thaler J. D. Achari Y. Lu T. Shrive N. G. Hart D. A. (2014). Estrogen receptor beta and truncated variants enhance the expression of transfected MMP-1 promoter constructs in response to specific mechanical loading. Biol. Sex. Diff 27, 14. 10.1186/s13293-014-0014-6 PMC4306124 25625008

[B238] Tsuboi K. Nagatomo T. Gohno T. Higuchi T. Sasaki S. Fujiki N. (2017). Single CpG site methylation controls estrogen receptor gene transcription and correlates with hormone therapy resistance. J. Steroid Biochem. Mol. Biol. 171, 209–217. 10.1016/j.jsbmb.2017.04.001 28412323

[B239] Turner H. McManus R. Kelly P. (2022). What are the effects of posterior corrective surgery, with or without thoracoplasty, on pulmonary function in adolescent idiopathic scoliosis? A systematic review and meta-analysis. Glob. Spine J. 14, 910–924. 10.1177/21925682221133750 PMC10240605 36377069

[B240] Urrutia J. Diaz-Ledezma C. Espinosa J. Beven S. H. (2011). Lumbar scoliosis in postmenopausal women: Prevalence and relationship with bone density, age and body mass index. Spine (Phila Pa 1776) 36, 737–740. 10.1097/BRS.0b013e3181db7456 21178842

[B241] Urrutia J. Espinosa J. Diaz-Ledezma C. Cabello C. (2011). The impact of lumbar scoliosis on pain, function and health-related quality of life in postmenopausal women. Eur. Spine J. 20, 2223–2227. 10.1007/s00586-011-1829-z 21538207 PMC3229743

[B242] Vafaeian B. Adeeb S. El-Rich M. Dulai S. K. Jaremko J. L. (2019). Prediction of mechanical behavior of cartilaginous infant hips in Pavlik harness: A subject-specific simulation study on normal and dysplastic hips. J. Orthop. Res. 37, 655–664. 10.1002/jor.24213 30604892

[B243] van der Horst-Bruinsma I. E. de Vlam K. Walsh J. A. Bolce R. Hunter T. Sandoval D. (2022). Baseline characteristics and treatment response to ixekizumab categorised by sex in radiographic and non-radiographic axial spondylarthritis through 52 Weeks: Data from three Phase III randomised controlled trials. Adv. Ther. 39, 2806–2819. 10.1007/s12325-022-02132-2 35429281 PMC9123018

[B244] Van Es L. J. M. van Royen B. J. Oomen M. W. N. (2022). Clinical significance of concomitant pectus deformity and adolescent idiopathic scoliosis: Systematic review with best evidence synthesis. N. Am. Spine Soc. J. 11, 100140. 10.1016/j.xnsj.2022.100140 35814492 PMC9256832

[B245] Vandenput L. Johansson H. McCloskey E. V. Liu E. Akesson K. E. Anderson F. A. (2022). Update of the fracture risk prediction tool FRAX: A systematic review of potential cohorts and analysis plan. Osteoporos. Int. 33, 2103–2136. 10.1007/s00198-022-06435-6 35639106

[B246] VanderJagt K. Butler M. G. (2019). Ehlers-Danlos syndrome and other heritable connective tissue disorders that impact pregnancies can be detected using next-generation DNA sequencing. Arch. Gynecol. Obstet. 3000, 491–493. 10.1007/s00404-019-05226-5 PMC8034485 31250196

[B247] Vescini F. Chiodini I. Falchetti A. Palermo A. Salcuni A. S. Bonadonna S. (2021). Management of osteoporosis in men: A narrative review. Int. J. Mol. Sci. 22, 13640. 10.3390/ijms222413640 34948434 PMC8705761

[B248] Wakkar A. Pati S. P. (2022). Assessment of knee and ankle proprioception during the third trimester of pregnancy and postpartum period among primiparous women: An observational longitudinal study. J. Educ. Health Promot 11, 241. 10.4103/jehp.jehp_311_22 36177431 PMC9514273

[B249] Walker J. M. (1980). Growth characteristics of the fetal ligament of the head of femur: Significance in congenital hip disease. Yale J. Biol. Med. 53, 307–316. 7445537 PMC2595829

[B250] Wallace A. L. Hollinshead R. M. Frank C. B. (2002). Creep behavior of a rabbit model of ligament laxity after electrothermal shrinkage *in vivo* . Am. J. Sports Med. 30, 98–102. 10.1177/03635465020300012901 11799003

[B251] Walters M. R. Nemere I. (2004). Receptors for steroid hormones: Membrane-associated and nuclear forms. Cell Mol. Life Sci. 61, 2309–2321. 10.1007/s00018-004-4065-4 15378202 PMC11138745

[B252] Wang L. Wang Y. Zhang P. Song C. Pan F. Li G. (2022). Gut microbiota changes in patients with spondyloarthritis: A systematic review. Semin. Arthritis Rheum. 52, 151925. 10.1016/j.semarthrit.2021.11.002 34844732

[B253] Wang L. Zhang Y. Zhao S. Dong X. Li X. You Y. (2020). Estrogen receptors (ESRs) mutations in adolescent idiopathic scoliosis: A cross-sectional study. Med. Sci. Monit. 26, e921611. 10.12659/MSM.921611 32218412 PMC7101201

[B254] Wang Y. X. J. Griffith J. F. (2010). Effect of menopause on lumbar disk degeneration: Potential etiology. Radiology 257, 318–320. 10.1148/radiol.10100775 20959546

[B255] Wang Y. X. J. (2016). Menopause as a potential cause for higher prevalence of low back pain in women than in age-matched men. J. Orthop. Transl. 8, 1–4. 10.1016/j.jot.2016.05.012 PMC5987020 30035087

[B256] Weingart A. Rynecki N. Pereira D. (2022). A review of neuromuscular training and biomechanical risk factor screening for ACL injury prevention among female soccer players. Bull. Hosp. Jt. Dis. 80, 253–259. 36030444

[B257] Weir T. M. R. (1991). Recovery of a growing ligament following immobilization a rabbit model. Calgary, Alberta CANADA: MSc Thesis, University of Calgary.

[B258] Weis C. A. Grondin D. Vernon H. (2016). The effect of phase of menstrual cycle on joint mobility in the cervical spine and extremities in nulliparous women: A cross-sectional study. J. Manip. Physiol. Ther. 39, 393–400. 10.1016/j.jmpt.2016.05.002 27346859

[B259] Wenger D. Duppe H. Tiderius C. J. (2013). Acetabular dysplasia at the age of 1 year in children with neonatal instability of the hip. Acta Orthop. 84, 483–488. 10.3109/17453674.2013.850009 24171679 PMC3822134

[B260] Wild C. Y. Steele J. R. Munro B. J. (2012). Why do girls sustain more anterior cruciate ligament injuries than boys?: A review of the changes in estrogen and musculoskeletal structure and function during puberty. Sports Med. 42, 733–749. 10.1007/BF03262292 22784194

[B261] Williams J. S. Dunford E. C. MacDonald M. J. (2020). Impact of the menstrual cycle on peripheral vascular function in premenopausal women: Systematic review and meta-analysis. Am. J. Physiol. Heart Circ. Physiol. 319, H1327–H1337. 10.1152/ajpheart.00341.2020 33064553

[B262] Wright G. C. Kaine J. Deodhar A. (2020). Understanding differences between men and women with axial spondyloarthritis. Semin. Arthritis Rheum. 50, 687–694. 10.1016/j.semarthrit.2020.05.005 32521322

[B263] Wu X. Chen L. Lu W. He S. Li X. Sun L. (2022). Discovery of novel variants on the CHD7 gene: A case series of CHARGE syndrome. Front. Genet. 13, 852429. 10.3389/fgene.2022.852429 35938004 PMC9355507

[B264] Wu Z. Dai Z. Yuwen W. Liu Z. Qiu Y. Cheng J. C. Y. (2021). Genetic variants of CHD7 are associated with adolescent idiopathic scoliosis. Spine (Phila Pa 1776) 46, E618–E624. 10.1097/BRS.0000000000003857 33290368

[B265] Wuytack F. Begley C. Daly D. (2020). Risk factors for pregnancy-related pelvic girdle pain: A scoping review. BMC Pregnancy Childbirth 20, 739. 10.1186/s12884-020-03442-5 33246422 PMC7694360

[B266] Xiong Y. Cai M. Dong P. Chen H. He W. Zhang J. (2022). Joint together: The etiology and pathogenesis of ankylosing spondylitis. Front. Immunol. 13, 996103. 10.3389/fimmu.2022.996103 36325352 PMC9619093

[B267] Xu L. Xia C. Sun W. Qin X. Qiu Y. Zhu Z. (2017). Genetic polymorphism of NUCKS1 is associated with the susceptibility of adolescent idiopathic scoliosis. Spine (Phila Pa 1776) 42, 1629–1634. 10.1097/BRS.0000000000002167 28338576

[B268] Yan B. Lu X. Qiu Q. Nie G. Huang Y. (2020). Predicting adolescent idiopathic scoliosis among Chinese children and adolescents. Biomed. Res. Int. 2020, 1784360. 10.1155/2020/1784360 32766304 PMC7387995

[B269] Yu W. D. Panossian V. Hatch J. D. Liu S. H. Fireman G. A. (2001). Combined effects of estrogen and progesterone on the anterior cruciate ligament. Clin. Orthop. Relat. Res. 383, 268–281. 10.1097/00003086-200102000-00031 11210964

[B270] Zaina F. Cordani C. Donzelli S. Lazzarini S. G. Arienti C. Del Furia M. J. (2022). Bracing interventions can help adolescents with idiopathic scoliosis with surgical indication: A systematic review. Child. (Basel) 9, 1672. 10.3390/children9111672 PMC9688311 36360400

[B271] Zaydman A. M. Strokova E. L. Pahomova N. Y. Gusev A. F. Mikhaylovsky M. V. Shevchenko A. I. (2021). Etiopathogenesis of adolescent idiopathic scoliosis: Review of the literature and new epigenetic hypothesis on altered neural crest cells migration in early embryogenesis as the key event. Med. Hypotheses 151, 110585. 10.1016/j.mehy.2021.110585 33932710

[B272] Zebis M. Andersen L. L. Brandt M. Myklebust G. Bencke J. Lauridsen H. B. (2016). Effects of evidence-based prevention training on neuromuscular and biomechanical risk factors for ACL injury in adolescent female athletes: A randomised controlled trial. Br. J. Sports Med. 50, 552–557. 10.1136/bjsports-2015-094776 26400955

[B273] Zelenko Z. Aghajanova L. Irwin J. C. Giudice L. C. (2012). Nuclear receptor, coregulator signaling, and chromatin remodeling pathways suggest involvement of the epigenome in the steroid hormone response of endometrium and abnormalities in endometriosis. Reprod. Sci. 19, 152–162. 10.1177/1933719111415546 22138541 PMC3343132

[B274] Zhang S. Doudoulakis K. J. Khurwal A. Sarraf K. M. (2020). Developmental dysplasia of the hip. Br. J. Hosp. Med. 81, 1–8. 10.12968/hmed.2020.0223 32730146

[B275] Zhou Z. Chen C. Zhang J. Ji X. Liu L. Zhang G. (2014). Safety of denosumab in postmenopausal women with osteoporosis or low bone mineral density: A meta-analysis. Int. J. Clin. Exp. Pathol. 7, 2113–2122. 24966919 PMC4069896

[B276] Zhu J. March L. (2022). Treating osteoporosis: Risks and management. Aust. Presc 45, 150–157. 10.18773/austprescr.2022.054 PMC9584792 36382174

[B277] Zonobi D. Hareendranathan A. Mostofi E. Mabee M. Pasha S. Cobzas D. (2018). Developmental hip dysplasia diagnosis at three-dimensional us: A multicenter study. Radiology 287, 1003–1015. 10.1148/radiol.2018172592 29688160

